# Significance of Pulmonary Endothelial Injury and the Role of Cyclooxygenase-2 and Prostanoid Signaling

**DOI:** 10.3390/bioengineering10010117

**Published:** 2023-01-14

**Authors:** Rosa Nickl, Sandra Hauser, Jens Pietzsch, Torsten Richter

**Affiliations:** 1 Department of Anesthesiology and Critical Care Medicine, University Hospital Carl Gustav Carus Dresden, Technische Universität Dresden, Fetscherstr. 74, 01307 Dresden, Germany; 2 Department of Radiopharmaceutical and Chemical Biology, Institute of Radiopharmaceutical Cancer Research, Helmholtz-Zentrum Dresden-Rossendorf, Bautzner Landstrasse 400, 01328 Dresden, Germany; 3 Faculty of Chemistry and Food Chemistry, Technische Universität Dresden, Mommsenstrasse 4, 01062 Dresden, Germany

**Keywords:** acute respiratory distress, cyclooxygenases, eicosanoids, endothelial barrier dysfunction, lung endothelium, lung infection, lung inflammation, prostanoid receptors, selective cyclooxygenase-2 inhibitors (COXIBs), traditional non-steroidal anti-inflammatory drugs (tNSAIDs)

## Abstract

The endothelium plays a key role in the dynamic balance of hemodynamic, humoral and inflammatory processes in the human body. Its central importance and the resulting therapeutic concepts are the subject of ongoing research efforts and form the basis for the treatment of numerous diseases. The pulmonary endothelium is an essential component for the gas exchange in humans. Pulmonary endothelial dysfunction has serious consequences for the oxygenation and the gas exchange in humans with the potential of consecutive multiple organ failure. Therefore, in this review, the dysfunction of the pulmonary endothel due to viral, bacterial, and fungal infections, ventilator-related injury, and aspiration is presented in a medical context. Selected aspects of the interaction of endothelial cells with primarily alveolar macrophages are reviewed in more detail. Elucidation of underlying causes and mechanisms of damage and repair may lead to new therapeutic approaches. Specific emphasis is placed on the processes leading to the induction of cyclooxygenase-2 and downstream prostanoid-based signaling pathways associated with this enzyme.

## 1. Introduction

The understanding and the importance of the endothelium has changed from a simple layer of cells lining the blood vessels to a significant complex network with countless fundamental functions. Its prominent involvement in the interplay of hemodynamic, humoral and especially inflammatory regulatory circuits has already led to a better understanding of the development and progression of various diseases, e.g., asthma, chronic obstructive pulmonary disease (COPD) [1] and acute respiratory distress syndrome [2]. Endothelial cells (EC) are in constant interaction with the surrounding blood stream, neighboring cells or the extracellular space, and fulfill both paracrine and endocrine functions. Therefore, the properties and appearance of endothelial cells are very heterogeneous. The layer thickness of the endothelium varies from 0.1 µm to 1 µm [3]. In this context, the heterogeneity of EC is due to a variety of requirements related to different tissues (such as lung, skin, or brain) and vessel types therein, such as arteries, veins, or capillaries. The diversity and especially the regional specialization of the cells is based on the transcriptional profile of the individual cell groups and depends on the tissue and the surrounding structures [4,5]. Non-fenestrated endothelial layers are mainly found in the macrovascular system, as well as in the capillary system of some organs, including the heart, lung, skin or brain. The primary function of this continuous cell layer type is to provide a barrier function. In contrast, there are continuously arranged fenestrated endothelial cells as in renal glomeruli or discontinuously arranged cell layers disrupted by pores, e.g., in liver, spleen or bone marrow. Thus, cell migration and selective permeability can be mediated. The high endothelial plasticity is a result of the interplay of a variety of phenotypic influences (e.g., inflammation, trauma, aging) on the one hand, and molecular features, such as the expression of a wide variety of mediators, on the other [6,7].

Two alveolar endothelial cell subtypes have been subsequently classified.

General capillary cells are bipotent progenitor cells, replenishing alveolar endothelium during maintenance and repair. They are positioned in contact with stromal cells and regulate vasomotor tone. They may give rise to so-called aerocytes, which are the second part of the alveolar endothelial. These large complex cells extend several alveoli and form multicellular tubes. They are closely fused with AT1 cells and form extremely thin regions of the gas exchange surface [8]. These highly specialized cells exist only in the lungs and are also a major contributor to leukocyte trafficking. The alveolar membrane is continuously exposed to a wide variety of airborne pathogens and toxins. This can have far-reaching consequences for the integrity of this delicate complex consisting of alveolar epithelial cells, intermediate basement cells, and capillary endothelial cells. Endothelial dysfunction and endothelial damage resulting from viral, bacterial, and fungal infections, mechanical stress, or aspiration are increasingly recognized as a major health problem. Understanding the underlying mechanisms of damage and repair is of great importance for the development of new compelling therapeutic approaches.

In particular focus in this review are the cyclooxygenase-2 and prostanoid-mediated signaling pathways. A greater understanding of the functional expression of this enzyme in the lung endothelium during the course of lung disease during therapy and in the healing process may allow important differential diagnostic and therapeutic conclusions. Experience in recent years with anti-inflammatory treatments targeting COX-2 through selective COX-2 inhibitors, also called COXIBs, has shown that sole and sustained inhibition of the enzyme is not always clinically indicated. It is often important to modulate the activity of COX-2 to the right extent and in the right time window, an aspect whose biochemical basis is also considered in this work.

In this review article, we summarize recent developments. A PubMed database search was performed in the 2nd quarter of 2022 using key words “acute lung injury”, “acute respiratory distress syndrome”, “aspiration”, “cyclooxygenase”, “endothelium”, “infection (bacterial, mycotic/fungal and viral)”, “lung” and “ventilator induced lung injury (VILI)” linked to each other by AND and to the phrases “cancer”, “extracorporal circulation”, “transplantation”, “cardiothoracic surgery” with NOT as Boolean function.

The abstracts of the identified studies were reviewed; all studies that met a priori exclusion criteria were excluded, and the full text was examined for those remaining.

## 2. Pulmonary Endothelial Dysfunction as a Result of Infections

Lower respiratory tract infections remain one of the leading cause of death worldwide, regardless of age [9]. Pulmonary infection is the primary cause in up to 51% of patients with acute lung injury (ALI) [10]. Research on infection-induced alveolar damage has largely focused on the mechanisms and pathophysiological processes related to the epithelial cells of the lung.

### 2.1. Bacterial Infections

Endotoxin or lipopolysaccharide (LPS), a proinflammatory mediator derived from the cell envelope of Gram-negative bacteria, compromises EC barrier function in vitro and in vivo primarily through the activation of toll-like receptor 4 (TLR-4) by activating neutrophils, macrophages and other cells producing proinflammatory mediators and free radicals [11,12,13,14]. A central role is played by the activation of the pulmonary nuclear factor kappaB (NF-κB), followed by the increased synthesis and release of cytokines, such as tumor necrosis factor alpha (TNF-α). This, in turn, activates neutrophils and increases their expression of the adhesion molecule E-selectin, while macrophages respond by synthesizing and releasing additional cytokines (Interleukine-1 (IL-1), IL-6, IL-8, IL-10) and TNF-α [15,16]. During LPS-induced ALI, prolonged sequestration time and arrest-like dynamic behavior of neutrophils have been shown to lead to neutrophil entrapment in capillaries and arterioles [17]. The neutrophil entrapment is followed by permeability changes of the lung endothelial cells and edema formation [18,19]. Furthermore, peeling of the endothelial glycocalyx from lung EC occurs [20]. This process of shedding leads to the release of syndecan-1 and presumably acts as an adaptation to inflammation by limiting damage by restructuring and loosening intercellular junctions [21]. LPS-induced disruption of the EC barrier is critically dependent upon changes in the cytoskeleton of lung EC, including the activation of the rat sarcoma homologue (Rho) signaling, tyrosine kinase(s) and protein kinase C (PKC) pathways [22,23,24]. This triggers contraction, and, thus, the subsequent opening of paracellular avenues of permeability [25,26,27]. Different histone deacetylase (HDAC) isoforms are also involved in the LPS-induced regulation of the cytoskeleton structures and, therefore, EC barrier disruption through deacetylation mechanisms [28,29]. In vitro investigation of specific HDAC inhibitor has already been demonstrated to mitigate LPS-induced impairment of human lung EC; thus, it may further come into focus as a future novel targeted therapy [30]. Early inhibition of heat shock protein 90 (Hsp90), and concomitant disruption of the Rho signaling appears to prevent LPS-induced hyperpermeability and may potentially provide another approach for further research efforts on targeted treatment [24].

Lipoteichoic acid (LTA) and peptidoglycans are components of the cell wall of Gram-positive bacteria. Their inflammation-inducing pathway leads via TLR-2. It occurs through the activation of the myeloid differentiation primary response 88 (MyD88) signaling pathway, immediately resulting in NF-κB activation and cytokine transcription [31,32]. In vitro experiments indicate increased permeability through a mechanism mediated by reactive oxygen and nitrogen species in lung endothelial cells after LTA exposure, which are released by alveolar macrophages and other cross-talking lung cells [33]. Furthermore, bacterial infection leads to inflammatory processes of the pulmonary endothelium not only by cellular components, but also by various toxins (Table 1).

### 2.2. Viral Infections

The impact of the SARS-CoV-2 pandemic brought viral respiratory diseases back into the focus of research efforts. The disintegration of profound vascular functions characterizes the similar clinical appearance of fulminant viral infections: edema, hemorrhage, thrombosis, and organ hypoperfusion. While the SARS-CoV-2, Dengue, and Ebola viruses affect various cell types, endothelial cells are the major target for Hanta, paramyxo, and Influenza A, H5N1 viruses [49,50,51,52,53]. Viral attachment is mediated by viral surface proteins that bind to specific glycan or protein cell receptors, resulting in cellular and host specificity [54]. Nonspecific attachment via interactions with scavenger receptors or various carbohydrates has also been reported [55]. In viral infections such as H1N1 [53], and presumably in SARS-CoV-2 [56], lung endothelial cells are responsible for regulating the immune response, which consists of the recruitment of the innate immune cells and the production of innate chemokines and cytokines. Depending on the type of virus, release of a wide variety of cytokine groups occurs. However, a common feature of all recent pandemic viruses is that they induce an excessive early cytokine response [57,58]. In response to cytokines, EC produces enzymes that trigger glycocalyx detachment, leading to disruption of endothelial barrier function. Dengue non-structural protein 1 (NS1) directly induces glycocalix shedding of lung endothelium through the upregulation of cathepsin L, endothelial sialidases, and heparanase through TLR-4, similar to bacterial infection via LPS [59]. However, in *Hanta* pulmonary syndrome, dramatic disruption of vascular barrier function is mediated by direct viral manipulation of inter-cellular junctions. Surface proteins increase vascular endothelial growth factor (VEGF)-dependent vascular permeability through the inactivation of β3-integrins. Induction of the RhoA signaling pathway via the Hantavirus N protein causes an additive VEGF-independent enhancement of permeability [60]. However, other cells of the immune defense system may play distinct roles in the interaction with endothelial cells in various viral infections. In a mouse model of experimental *human Metapneumovirus (hMPV),* macrophages are required for early entry and replication in the lung. Therefore, alveolar macrophages appear to function as regulatory cells that promote clinical disease, airway obstruction, and general lung pathology [61]. These results contrast with other studies of RSV infection, belonging to the same Paramyxoidae family as hPMV. The role of macrophages, being exposed to viral pathogens of RSV, is to contribute to the antiviral innate immune response [61,62].

Secondary bacterial infection in the course of pneumonia initially caused by viruses is associated with increased mortality and morbidity rates [63,64]. The viral-bacterial interplay causes a fatal enhanced pro-inflammatory innate immune response in pulmonary EC [65]. In Influenza pneumonia, but especially in COVID-19, bacterial co-infection dramatically increases the mortality rate up to 75.9% [64]. Further mechanisms of lung endothelial damage elicited by variant virus species are shown in Table 2.

### 2.3. Mycotic Infections

Another dreaded disease is pulmonary mycotic infection. Invasive mycoses are a severe hazard and a major cause of morbidity and mortality, especially in immunocompromised patients. Invasive aspergillosis (IPA) is one of the most serious opportunistic infections, with mortality rates ranging from 35% to over 60%, and plays a particularly significant role in neutropenic or bone marrow transplant patients [78,79]. However, invasive fungal infection can also occur in immunocompetent patients. The host inflammatory response and virulence here depend, inter alia, on the composition of the fungal cell wall [80,81,82,83]. For example, the chitin components of *Candida albicans* induce IL-1β synthesis through macrophages [83]. The angioinvasive behavior of fungal pathogens leads to tissue destruction and local vascular thrombosis, thus enabling the fungus to invade tissues and spread hematogenously to other organs [84]. Hyphae of *Aspergillus fumigatus* stimulate EC to express the leukocyte adhesion molecules E-selectin, tissue factor and vascular cell adhesion molecule 1 (VCAM-1) and secrete the proinflammatory cytokines TNF-α and IL-8 [85]. *Candida* additionally induces the expression of ICAM-1 [86]. *Aspergillus fumigatus* is able to stimulate endothelial cells to express tissue factor, thus triggering and aggravating thrombosis [87]. A unique feature of *Aspergillus* and *Zygromycetes* species is the ability to carry out luminal as well as abluminal angioinvasion [88,89]. In this regard, abluminal *Aspergillus* infection causes greater expression of these procoagulant and proinflammatory genes (except IL-8) while causing less endothelial cell damage [90]. *Candida* and *Aspergillus spp.* induce various cell-damaging reactions, which are referred to as PANoptosis in their entirety. Several caspases (1,3,7 and 8), as well as mixed lineage kinase domain such as pseudokinase (MLKL) phosphorylation, are involved in this Z-DNA-binding protein-dependent activation leading to pyroptosis, necroptosis and apoptosis [91]. In IPA, toxic products from activated neutrophils additionally exacerbate fungus-induced tissue necrosis in non-neutropenic hosts [85,92]. Table 3 shows further exemplary pathogens of various invasive fungi. Some fungal species do not interact directly with the lung endothelium but use a Trojan horse transport mechanism to enter the bloodstream. For example, *Cryptococcus neoformans* [93] and *Histoplasma capsulatum* [94] induce their own phagocytosis through mononuclear cells of the immune system, e.g., neutrophils and macrophages. They can proliferate therein and use these host cells as shuttles into the vasculature. *Cryptococcus neoformans* can spread further through a damaged lung epithelium.

## 3. Pulmonary Endothelial Dysfunction as a Result of Mechanical Stress and Aspiration

In addition to infectious causes, mechanical stress is often another trigger for pulmonary endothelial damage. The epitome of such a burden is the mechanical ventilation of patients especially in pulmonary disease. Mechanical stress of the lung parenchyma is a major factor determining ventilator-induced lung injury (VILI). Overdistension of the lung tissue results in the release of proinflammatory substances that further damage the lung and escalate the progression of the inflammatory response, contributing significantly to mortality [108]. Excessive alveolar stretch results in decreased barrier function of EC due to rearrangement of the cytoskeleton and progressive formation of paracellular gaps [109]. Increased expression of signaling and contractile proteins in pulmonary EC, including Rho GTPase, myosin light chain, myosin light chain kinase, zipper-interacting protein kinase, protease-activated receptor 1, caldesmon, and Hsp27 [110] and high-mobility group Box 1 (HMGB1) [111], leads to destabilization of cell–cell junctional cadherins, loss of peripheral organized actin fibers, and the development of central stress fibers due to mechanical stretch [112]. Disruption of endothelial-specific VE cadherin bonds is a central mechanism of altered pulmonary endothelial barrier function [113,114]. There is evidence that this mechanism enables transendothelial migration of leukocytes [115]. Presumably, this results in the local accumulation of leukocytes and platelets in microvessels. Loss of endothelial barrier function is a central pathophysiological mechanism in the development of ALI. The increased permeability of microvascular barriers leads to extravascular accumulation of protein-rich fluid in the interstitium and air spaces [116,117]. Another pathophysiological process of VILI is the activation and phosphorylation of NF-kB, triggering the activation of inflammatory cells and the formation of a chemoattractant gradient, inducing an inflammatory cascade [118,119]. The ongoing upregulation of proinflammatory cytokines in EC, including MCP-1, IL-6 and IL-8, promotes further local and systemic inflammation [120,121]. The mechanoreceptor TRPV4 (transient receptor potential cation channel subfamily V member 4) plays another key role in the development of VILI and, according to recent studies, induces pulmonary EC barrier disruption by disrupting mitochondrial bioenergetics [122]. VILI is opposed by macrophage TLR 4, which is involved in the recovery and resolution of VILI. TLR4 activated by Hsp70 conditionally promotes macrophage efferocytosis by suppressing the shedding of Mer receptor tyrosine kinase by inactivating ADAM17 signaling [123]. Another way to reduce ventilator-induced mechanical stress on pulmonary EC is through the concept of protective ventilation [124].

Hence, pulmonary endothelium is becoming the focus of scientific therapeutic approaches for the treatment of acute lung injury. One promising therapeutic option targeting the endothelium are ligands that bind to receptors on EC and activate stabilizing intracellular pathways, mediating cytoskeletal reorganization and tightening of the VE cadherin bonds. One approach to strengthen the endothelial barrier appears to be the lipid sphingosine-1-phosphate (S1P). This binds to specific endothelial receptors and induces cytoskeletal reorganization, activation of the Ras-related C3 botulinum toxin substrate (Rac), and promotes the formation of adherence junctions [125,126]. However, some S1P receptors can also trigger destabilizing activities depending on the time and duration of administration [127]. Another stabilizing agonist is the active fragment of the Robo4 ligand Slit (Slit2N), which inhibits tyrosine phosphorylation of VE cadherin [128]. Thus, the internalization of VE cadherin is suppressed and the loss of integrity of the endothelial barrier through proinflammatory cytokines is prevented [129]. Other stabilizing agonists, such as angiopoietin 1 and atrial natriuretic peptide, have been identified and require further investigation, particularly with regard to isolated endothelial effects [130].

Another significant mechanism of inflammatory damage to lung endothelium is aspiration that potentially causes chemical pneumonitis and pneumonia. Aspiration of gastric contents is considered an important risk factor for the development of ARDS. The hallmark of the inflammatory response to gastric acid is characterized by the acute infiltration of neutrophils in the alveolar space, alveolar hemorrhage, intraalveolar and interstitial edema [131]. Low pH acid aspiration primary injures the airway and alveolar epithelium but also results in endothelial injury [132]. Tumor necrosis factor α plays an essential role in this process. It leads to leukocyte activation and induces, among other things, the expression of endothelial adhesion molecules that lead to the migration of neutrophils into the alveoli [133]. The proinflammatory cytokine IL-8 is also essential in this process as a chemotactic for neutrophils and promotes migration through activated endothelium and activation of neutrophils [134]. It must not be neglected that gastric contents contain various other substances such as food residues, bacteria and their constituents, but also cytokines such as IL-1β. Therefore, all these factors tend to lead to complex inflammatory damage mechanisms in the pathogenesis of aspiration-induced lung injury [135,136]. Seawater, similar to the gastric content, is a hyperosmolar mixture. In addition to bacteria and viruses, it contains a high level of calcium and sodium and has a lower temperature [137]. Seawater aspiration primarily affects the regulation of pulmonary surfactant [138] and the alveolar epithelium directly [139]; furthermore, it leads to inflammatory reactions and destabilization of the endothelial barrier in the lungs. Endothelial semaphorin 7A promotes the inflammation and edema by increasing the endothelial permeability in seawater aspiration-induced ALI [140]. The expression of hypoxia-inducible factor 1 α (HIF-1α) is also upregulated due to seawater stimulus [140]. Another edema promoting factor is the increased expression of endothelial aquaporin 1 (AQP1), indicating elevated water permeability of the blood–air barrier [141]. 

The previous summary shows the multifactorial pathogenesis and signaling cascades that could be involved in inflammatory damaging processes of the pulmonary endothelium. In the next section, we will focus on the various COX signaling pathways that play a significant role in these processes.

## 4. Cyclooxygenase Signaling Pathways

### 4.1. Cyclooxygenases, Prostanoids, Prostanoid Receptors, and Downstream Signaling

**Figure 1:**
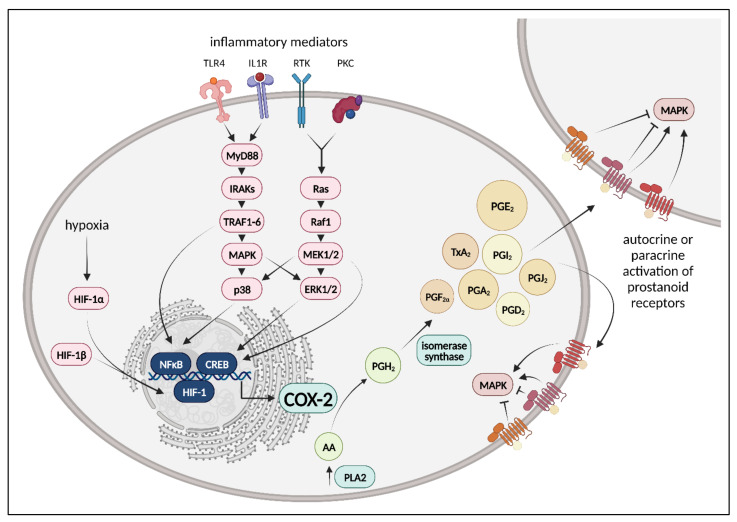
Schematic depiction of selected cyclooxygenase-2 (COX-2) signaling pathways. Ligand binding of inflammatory mediators to their receptors, such as pattern recognition receptors (Toll-like receptor 4, TLR-4), cytokine receptors (interleukine-1 receptor, IL1R), tyrosine kinase receptors (RTK) or protein kinase C (PKC) activates inflammatory signaling via myloid differentiation primary response gene 88 (MyD88)- or rat sarcoma (Ras)-dependent activation of several mitogen-activated protein kinases (MAPK), such as extracellular signal-related kinases 1/2 (ERK1/2) or p38. Thereby, activation of proinflammatory transcription factor nuclear factor κB (NF-κB) or cAMP response element-binding protein (CREB) is mediated. Further, hypoxia induces the activation of hypoxia-inducible factor-1α (HIF-1α) leading to the transcription of HIF-regulated genes. COX-2 is regulated by NF-κB, CREB, or HIF-1α, amongst others; therefore, it is expressed under proinflammatory or hypoxic conditions. COX-2 metabolizes arachidonic acid (AA), which is mobilized from the sn-2 position of membrane phospholipids through phospholipase A_2_ (PLA_2_) in a two-step reaction into prostaglandin H_2_ (PGH_2_), a substrate for several downstream isomerases and synthases in the generation of prostanoids: prostaglandins D_2_ (PGD_2_), E_2_ (PGE_2_), F_2α_ (PGF_2α_), A_2_ (PGA_2_), and J_2_ (PGJ_2_), thromboxane A_2_ (TXA_2_) and prostacyclin (PGI_2_). The size of the prostanoids shown corresponds to their relevance for inflammatory processes in the lung endothelium. Prostanoids act in an autocrine or paracrine manner by binding specific prostanoid receptors, which are class A, rhodopsin-like G protein-coupled receptors. Prostanoid receptors induce both classical G protein-dependent pathways and G protein-independent pathways, whereas downstream signaling is shaped by the prostanoid ligand and either activates or inhibits MAPK signaling. Mitogen-activated protein kinase kinase 1/2, MEK1/2; interleukine-1 receptor associated kinases, IRAKs; rapidly accelerated fibrosarcoma 1, Raf1; tumor necrosis factor receptor associated factor 1, TRAF1-6. Created with BioRender.com.

Eponymous and central enzymes of the cyclooxygenase (COX) signaling pathways are the cyclooxygenases (COX), which are also known as prostaglandin G/H synthases. They catalyze the first two steps in the biosynthesis of prostanoids, well-known key players in the onset and resolution of inflammation, starting from arachidonic acid (AA), which is mobilized from the sn-2 position of membrane phospholipids via phospholipase A2 [142,143,144,145]. COX exists in three distinct isoforms: COX-1, COX-2, as well as a splice variant of COX-1, COX-3, which lacks enzymatic cyclooxygenase activity [146]. COX-1 is constitutively expressed in most tissues, compared to COX-2, which is regulated by transcription factors such as nuclear factor of activated T cells (NFAT), cAMP response element-binding protein (CREB), NF-κB, or hypoxia-inducible factors (HIF); therefore, it is induced by proinflammatory stimuli, cytokines, mitogens, activated platelets, as well as hypoxia and is constitutively expressed only in certain parts of the forebrain, cortex, hippocampus, and parts of the kidney [147]. Both COX-1 and COX-2 metabolize AA into prostaglandin G2 (PGG2) through their cyclooxygenase activity, followed by conversion of PGG2 into prostaglandin H2 (PGH2) via their hydroperoxidase activity. PGH2 then serves as a substrate for several downstream isomerases and synthases, whose expression depends on the cell type and is coupled to the COX isoform, in the generation of prostanoids: prostaglandins D2 (PGD2), E2 (PGE2) and F2α (PGF2α), thromboxane A2 (TXA2) and prostacyclin (PGI2) [143]. Dehydration also gives rise to the cyclopentane ring of PGE2 and PGD2 the cyclopentenone prostaglandins PGA2 and PGJ2 [148]. Prostanoids are synthesized in almost all mammalian tissues and regulate important homeostatic functions, such as the maintenance of the cardiovascular function, hemostasis or gastric epithelial cytoprotection [149,150]. In general, COX-2 exerts a dual role in the onset and resolution of inflammation, as evidenced by the fact that COX-2 expression peaks first at the onset of inflammation (within 2 to 6 h after stimulus) and second during the resolution phase (usually within 24 and 48 h). Moreover, both peaks coincide with the maximum PGE2 and PGD2 expression, respectively, indicating that COX-2 is proinflammatory during the early stage of inflammation, but acts in inflammation resolution at later stages [151]. Selected COX-2 signaling pathways are shown in Figure 1.

Prostanoids exert their function in an autocrine or paracrine manner by binding specific prostanoid receptors, which are classified into the five basic types DP, EP, FP, TP, and IP, for the PGD2, PGE2, PGF2α, TXA2, and PGI2 receptors, respectively, referring to their preferred binding partner. Prostanoid receptors belong to the class A, rhodopsin-like G protein-coupled receptors. Ligand binding induces both classical G protein-dependent pathways and G protein-independent pathways. Prostanoid receptors are promiscuous and often couple to more than one G protein; thereby, these trigger different signaling pathways. Further, G protein-independent signaling via a β-arrestin/Src complex, resulting in activation of the epidermal growth factor receptor (EGFR) and downstream Akt signaling, was shown for EP2 and EP4 receptors. However, prostanoid receptors function according to the principle of ligand-induced selective signaling; hence, downstream signaling is shaped by the prostanoid ligand. Therefore, another classification of prostanoids can be undertaken according to their influence on MAP kinase: MAP kinase inhibition through cAMP-dependent pathways (PGI2 and PGD2), MAP kinase activation (PGF2α and TXA2), and both, depending on the subtype of receptor bound (PGE2) [152,153,154,155,156]. The expression of all prostanoid receptors discussed above, except of DP2, has been shown in endothelial cells [157,158,159,160,161,162].

In addition to some prostanoids, low-dose oxidized phospholipids, such as oxidized 1-palmitoyl-2-arachidonoyl-sn-glycerol-3-phosphorylcholine (OxPAPC), can bind to EP4 receptor, which plays a central in promoting or disrupting the endothelial barrier function. In this regard, low-dose OxPAPC has enhancing characteristics on pulmonary endothelial barrier function, when sensed by the EP4 receptor, by tightening cell junctions and remodeling the cytoskeleton of EC [163]. Higher doses have the contrary effect, leading to disruption [164]. These opposite effects have also been experienced with PGE2, depending on the relative expression of the receptor subtype [165]. PGD2 was also found to have promoting properties on pulmonary endothelial barrier function in human pulmonary microvascular cells [166], whereas only moderate to no effects on lung endothelial cell integrity were demonstrated for human pulmonary arterial EC [167]. 

### 4.2. COX Pathways in Healthy and Injured Lung Endothelium

#### 4.2.1. COX Pathways in Healthy Lung Endothelium

Within the healthy blood vessel system, COX-1 is expressed in the endothelium where it couples with prostacyclin synthase to produce vascular and circulating antithrombotic PGI2, counteracting platelet COX-1-derived TXA2 [147,168,169]. In contrast, COX-2 is only slightly expressed constitutively in endothelial cells of the most blood vessels; however, these few constitutively expressed COX-2 molecules actually exert cardioprotective effects, as underlined by the severe cardiovascular side effects of selective COX-2 inhibitors (COXIBs) in long-term use [147]. Further, pulmonary endothelium-derived prostacyclin and prostaglandins contribute to pulmonary vasodilation at birth, thereby preventing the development of a persistent pulmonary hypertension of the newborn [170].

#### 4.2.2. COX Pathways in Injured Lung Endothelium

Many studies show overexpression of COX-2 and the resulting increased production of prostanoids during an inflammatory reaction in the lung, usually focusing on lung epithelial cells [171], fibroblasts, or inflammatory cells [172] (reviewed in [173,174,175,176]). For example, in a rat model of meconium aspiration, COX-2 expression has been induced in the respiratory epithelium and in alveolar macrophages [177]. In general, macrophages represent a major source of COX-2 and downstream signaling molecules in inflammatory conditions of the lung. While some studies have investigated prostanoids and downstream signaling, COX-2 expression in the lung endothelium in inflammatory situations is less known. 

##### Influence of Infections (Bacterial, Viral, Mycotic) on COX Pathways

LPS, the prototypical bacterial pathogen-associated membrane pattern (PAMP) and a major component of the outer cell membrane of Gram-negative bacteria, for example *Haemophilus influenzae, Pseudomonas aeruginosa, Bordetella pertussis*, or *Legionella pneumophila,* is known to induce the expression of COX-2 via an increase in phosphorylated p38 MAPK and a biphasic, glutathione-dependent activation of p42/44 MAPKs [178], as well as through the activation of NF-κB [179]. However, Gram-positive bacteria are also able to induce the upregulation of COX-2 and PGE2 in lung endothelial cells and alveolar macrophages via activation of MAPK, as shown by Szymanski et al. using a *Streptococcus pneumoniae* ex vivo infection model of human lung tissue [180]. *Streptococcus pneumoniae* further induces COX-2 in alveolar macrophages, thereby contributing to exacerbating fibrosis in a mouse model [181].

SARS-CoV-2 virus infections upregulate COX-2 expression, thereby, strengthening lung inflammation and injury observed in COVID-19 patients [182]. Further, hypoxia in severe COVID-19 may initiate the COX/thromboxane pathway in endothelial cells, leading to vasoconstriction and increased probability of thrombotic events [183]. Other viruses, such as RSV, HPIV-3 or H5N1, upregulate COX-2 in bronchial and bronchiolar epithelial cells and macrophages [184,185,186,187]. In a very recent paper, Gopalakrishnan showed that influenza infection induced COX-2 expression in inflammatory macrophages, which contributes significantly to influenza-induced lethality [188]. In contrast, poly(I:C), a viral PAMP that can be considered a synthetic analogue of double-stranded RNA present in, for example, reoviruses, which activates TLR-3, does not enhance COX-2 expression in mouse lung tissue. COX-2 knockout mice showed enhanced anti-poly(I:C) interferon responses, suggesting that COX-2 inhibitors might be a potential anti-viral therapy via boosting of the endogenous anti-viral response when provided soon after infection [189]. 

In mycotic infections with *Candida albicans* or *Candida auris,* the activation of MAPK pathways and proinflammatory signaling has been recognized, paralleled by the fungal evasion of the innate immune response [190]. A contribution to this evasion is probably provided by the COX-2/PGE2 axis and subsequent signaling via IL-33 that negatively regulates immune responsiveness, as shown in mouse models of fungal exposure with *Aspergillus fumigatus* [191] or *Alternaria alternata* [192]. This has been confirmed by in vivo COX-2 inhibition in these models, as well as in a mouse model of challenge with *Histoplasma capsulatum* [193].

##### COX-Pathways and ARDS

ARDS manifests in acute respiratory failure and increased-permeability pulmonary edema and is caused by direct injury to the lung, such as pneumonia or aspiration, as well as indirect mechanisms, such as sepsis or burn [194]. ARDS is usually accompanied by refractory hypoxemia and patients need mechanical ventilation [2]. Accordingly, the lung endothelium in ARDS exhibits inflammatory activation and loss of barrier function, in part mediated via COX pathways [195,196]. In this regard, it has been shown that COX-2 and PGE2 are significantly upregulated in remote ARDS after burn injury, which is induced by the binding of substance P (SP) to neurokinin-1 receptor (NK1R) and signaling via ERK1/2 and NF-κB [197]. PGE2, PGI2, and PGA2 do potentially exert barrier-protective and anti-inflammatory effects on lung EC in vitro and in vivo, tested in vitro in response to thrombin (barrier-disruptive), IL-6, and LPS (proinflammatory), and in vivo in a mouse model of acute lung injury (challenge with LPS 1mg/kg intratracheally) [167]. Ohmura et al. confirmed the barrier-protective effects of PGA2 in response to thrombin or LPS treatment in human pulmonary EC and in vivo and elucidated its mechanism of action via EP4 receptor and activation of Ras-related protein 1/Ras-related C3 botulinum toxin substrate 1 (Rap1/Rac1) GTPase and PKA targets at cell adhesions and cytoskeleton: VE cadherin, p120 catenin, zona occludens-1 protein (ZO-1), cortactin, and vasodilator-stimulated phosphoprotein (VASP) [198]. A different study showed that PGE2 binding to EP1 and EP4 receptor disrupts the barrier between pericytes and endothelial cells [199], underscoring the contrasting actions of PGE2 depending on EP receptor type as well as subsequent G protein coupling. PGD2 has also been shown to enhance the barrier function of human pulmonary EC challenged by thrombin, with these effects being mediated mainly by EP4 rather than the DP1 receptor. This highlights the fact that prostanoids and prostanoid receptors act in a pleiotropic manner [166]. In contrast, TXA2 induces disruption of the lung endothelial barrier function via TP receptor-mediated increase in intracellular Ca^2+^ concentration and activation of Rho kinase, resulting in actin cytoskeletal rearrangement, as investigated in vitro using HPAEC, HMVEC, and HUVEC [200]. On the other hand, 15d-PGJ2 is another anti-inflammatory prostanoid activating antioxidant genes via transcription factor nuclear factor erythroid 2-related factor 2 (Nrf2), and is generated by COX-2 in the late phase of inflammation as shown in a mouse model of acute lung injury induced by carrageenin [201].

##### COX-Pathways and Hypoxia

Hypoxia, as a consequence of, for example, aspiration, is known to induce hypoxic pulmonary vasopression (HPV), intending to match ventilation with perfusion and optimize gas exchange. If hypoxia is global, a generalized HPV occurs and may lead to pulmonary arterial remodeling and hypertension. According to animal studies of different species, in the acute phase of HPV, COX-2 is primarily linked to TXA2 synthase; therefore, it contributes to pro-proliferative pulmonary vasoconstriction, whereas in chronic HPV, COX-2 seemed to be coupled to PGI2 synthase, thereby promoting anti-proliferative pulmonary vasodilation (reviewed in [202]).

**Figure 2:**
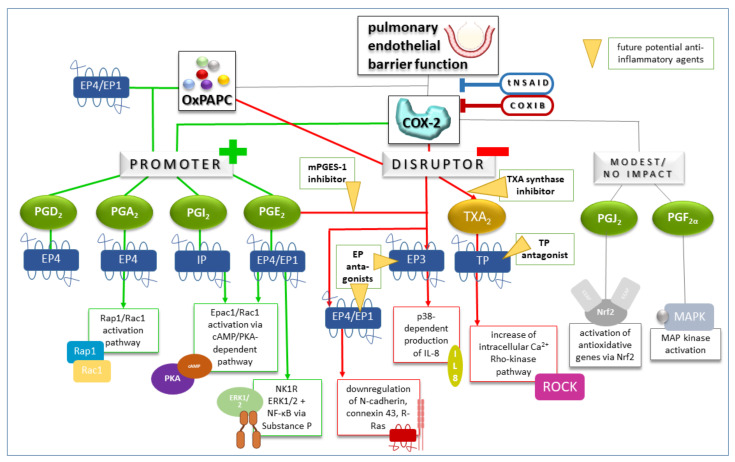
Determinants of pulmonary endothelial barrier function with a relationship to COX and present rsp. future therapeutic anti-inflammatory agents. Prostanoids as reaction products of the COX enzymes are presented as well as their subdivision into promoters and disruptors of the barrier function depending on the binding receptor and exemplary associated, with proven subsequent signaling pathways so far. Further, established and potential therapeutic anti-inflammatory agents and their targets are depicted. Cyclooxygenase, COX; selective inhibitors of COX-2, COXIB; exchange protein directly activated by cAMP 1, Epac1; prostaglandin E receptor 1, EP1; prostaglandin E receptor 3, EP3; prostaglandin E receptor 4, EP4; extracellular signal-regulated kinase 1/2, ERK1/2; interleukine 8, IL-8; prostacyclin receptor, IP; microsomal prostaglandin E 2 synthase-1, mPEGS-1; nuclear factor kappa B, NF-κB; neurokinin 1 receptor, NK1R; nuclear factor erythroid 2-related Factor 2, Nrf2; oxidized 1-palmitoyl-2-arachidonoyl-sn-glycerol-3-phosphorylcholine, OxPAPC; prostaglandin A2, PGA2; prostaglandin G2, PGD2; prostaglandin E2, PGE2; prostaglandin F2α, PGF2α; prostaglandin I2, PGI2; prostaglandin J2, PGJ2; protein kinase A, PKA; Ras-related protein 1, Rap1; Ras-related C3 botulinum toxin substrate 1, Rac1; traditional non-steroidal anti-inflammatory drugs, tNSAID; thromboxane receptor, TP; thromboxane A2, TXA2.

Another disease associated with hypoxia is the hepatopulmonary syndrome (HPS), which occurs in 15 to 30 % of patients with liver cirrhosis and is characterized by abnormal gas-exchange-induced hypoxia leading to intrapulmonary vasodilation, pulmonary vascular shunting and pulmonary angiogenesis. One major factor in the pathogenesis of HPS is assumed to be the accumulation of activated CD68+-macrophages in the lung microvasculature. Liu et al. found the COX-2/PGE2 signaling pathway to be activated in common bile duct ligation rat lung in vivo (an animal model of HPS) and PMVEC in vitro, which resulted in the accumulation of macrophages via an imbalance in the ratio of secreted bone morphogeneticprotein-2 (BMP-2) to crossveinless-2 (CV-2) [203]. The pathological pulmonary angiogenesis occurring in HPS, characterized by a collective directional migration of EC, is mediated by the activation of the PKC/Rac signaling pathway via the COX-2/PGE2 axis. This has been shown by Tang et al. in HPMVEC stimulated with HPS patient serum [204]. Figure 2 summarizes determinants of pulmonary endothelial barrier function with a relationship to COX and presents rsp. future therapeutic anti-inflammatory agents.

### 4.3. Therapeutic Approaches to Inhibit Cyclooxygenases in Lung Injury and Treatment Response

Unselective inhibition of cyclooxygenases through traditional non-steroidal anti-inflammatory drugs (tNSAIDs), such as, for example, aspirin, ibuprofen or diclofenac, is a long-established option to treat pain or inflammation. As tNSAIDs inhibit both the constitutive COX-1 and the inducible COX-2 they are, on long-term use, associated with gastric and renal side effects, mainly by disturbing the natural balance of prostanoids synthesized by COX-1. To overcome these problems, differently selective inhibitors of COX-2 (COXIBs) were designed based on structural differences between both COX isoforms: in its tertiary protein structure, COX-2 forms a side pocket, which COX-1 is lacking [205]. The majority of COXIBs, such as Celecoxib, Valdecoxib, and Rofecoxib, exhibit fewer gastric side effects compared to tNSAIDs, but increase the risk of cardiovascular side effects on long-term use [206]. This can be explained, amongst other concepts, by the disturbed balance of mainly COX-1-derived TXA2 and mainly COX-2-derived PGI2 towards thrombogenic TXA2, as well as increased metabolization of AA by 5-lipoxygenase into leukotrienes, established mediators of allergy and inflammation. Enhanced cardiovascular toxicity has even led to the withdrawal of rofecoxib and valdecoxib from the market [207,208]. Nevertheless, in the usage of tNSAIDs and COXIBs, the individual benefits and risks of the patients have to be considered. Especially in emergency and intensive care medicine, the advantages of COXIBs regarding their anti-inflammatory and analgesic effects often outweigh the risks. Celecoxib, etoricoxib, and parecoxib, a prodrug of Valdecoxib, are currently approved in Germany for the treatment of rheumatic diseases (e.g., such as arthritis and Bekhterev’s disease) and chronic pain, as well as for short-term postoperative pain management. In terms of inflammatory reactions in the lung, parecoxib is currently used in the treatment of ALI/ARDS and HPS.

With regard to potential further applications of COXIBs in lung inflammation, preclinical studies show beneficial effects of celecoxib or parecoxib administration. In a rat model of COPD, induced by nitrogen dioxide exposure, inhibition of COX-2 through celecoxib has been shown to reduce lung inflammation [209]. In a rat model of systemic inflammation induced by burn injuries, parecoxib applied intramuscularly after burn injury decreased plasma levels of inflammatory cytokines and lung myeloperoxidase level, and ameliorated systemic and lung inflammation [210]. Additionally, parecoxib could effectively reduce COX-2 expression, PGE2 level and lung injury induced by meconium aspiration in a rabbit model [211]. 

Patients receiving large volume and long-term mechanical ventilation due to, for example, lung injury or respiratory dysfunction are particularly susceptible to VILI, accompanied by local inflammation and lung injury up to systemic inflammation and multiple organ failure. Pharmacologic inhibition of COX-2 with CAY10404 significantly decreased COX activity and attenuated VILI in mice, attenuating barrier disruption and inflammation [212]. Meng et al. have confirmed this in a rat model of VILI, where parecoxib significantly improved gas exchange and survival by reducing edema, decreasing local and systemic inflammation and apoptosis [213]. Partly, this positive effect of COX-2 inhibition in VILI might be due to reduced recruitment of Ly6Chi monocytes that usually migrate to the site of lung injury and contribute to oxidative stress and inflammation [214]. Qin et al., recently showed in a rat model of lung ischemia-reperfusion injury (LIRI), which often occurs in pulmonary or cardiopulmonary surgeries and develops into ARDS, that a pretreatment with parecoxib ameliorates LIRI via reduction in oxidative stress and inflammatory mediators, and upregulation of heme oxygenase-1 in lung tissue [215]. 

In two different models of HPS, parecoxib was used to inhibit the activation of the COX-2/PGE2 signaling pathway, thereby, reducing the accumulation of activated CD68+-macrophages [203] as well as the directional migration of pulmonary EC [204], which are two major pathophysiological processes in the development of HPS.

Currently, cyclooxygenase inhibition through either tNSAIDs or COXIBs in order to reduce the cytokine storm and systemic inflammation in COVID-19 is a frequently investigated topic, and has to be discussed with regard to a potentially suppressed immune response due to COX-2 inhibition. Benefits for tNSAID use are likely, as they reduce the risk of development of severe disease in COVID-19 patients [216]. A recent study by Chen et al. showed that inhibition of COX-2/PGE2 signaling via ibuprofen and meloxicam, a moderately selective COX-2 inhibitor, had no effect on ACE2 expression, viral entry, or viral replication [182], thereby disproving the concerns that ibuprofen might lead to more severe infections via upregulation of ACE2. Based on a literature review, diclofenac (or other COX-2 inhibition medications) was considered recently for its anti-inflammatory and virus toxicity properties. For effectiveness and decreased risk, the use was recommended in high dose and in the early course of the disease (post-infection and/or symptom presentation) [217]. Recent evidence suggests to use of dual compounds COX-2 inhibitors/TP antagonist, providing anti-inflammatory effects and cardiovascular safety [218,219]. This was underlined by a clinical study indicating that adjuvant treatment with celebrex promotes the recovery of ordinary and severe COVID-19 [220].

## 5. Conclusions

Multiple factors, such as viral, bacterial and mycotic infections, aspiration or VILI, can severely damage the pulmonary endothelial tissue through different mechanisms, leading to consecutive hypoxemia and the development of ARDS. There is little diagnostic evidence to identify, for example, infection as a cause of pulmonary EC damage through inflammatory signaling pathways. Type and concentration ratios of the various prostanoid mediators involved in the inflammatory process or the extent of different up- and downstream signaling pathways cannot be determined analytically and a fortiori in the absence of clinical conditions, or only with great difficulty, to distinguish between tissue and organ-specific differences. However, it is precisely these differences that offer potential starting points for targeted individualized therapeutic intervention. An approach that serves to protect selected organs and tissues by modulating COX-2 activity and prostanoid signaling is indicated in addition to systemic anti-inflammatory therapy in many disease conditions, especially those described here. Such a targeted treatment is very difficult due to the complexity of the respective cellular interactions and specific microenvironmental conditions, especially in the presence of relevant comorbidities, and should be provided in the right time of the inflammatory process. Of course, the innate immune response and inflammatory processes are always an essential part of the healing process as well, and poorly balanced interventions can quickly lead to adverse effects. Therefore, each case must be handled individually and the type of drugs, possible concomitant drugs, dosage and duration of therapy has to be well-considered. In addition, there are approaches to investigate the anti-inflammatory effects of natural compounds. Components of pomegranate increased IL-10 gene expression and simultaneously decreased NO and TNF-α levels in LPS-exposed lung cells. However, the therapeutic potential of such compounds needs further investigation, specifically for acute lung endothelial injury [221]. Nevertheless, the development of new agents and the discovery and characterization of new drug targets will significantly advance this therapeutic approach. Promising approaches for selective inhibition of COX-2 include the use of hybrid or bifunctional compounds such as nitric oxide-releasing COXIBs. New treatment options should also be considered that allow for the local release of anti-inflammatory drugs via biomaterials, with the possibility of controlled optimal dosage and treatment duration as well.

## Figures and Tables

**Table 1:** Exemplary effects of various bacterial toxins on lung endothelial cells (EC).

	Toxin	Impact on EC	Sources
Gram positive			
*Staphylococcus aureus*	α-toxin	disruption of endothelial-cell tight junctions through (activating acid sphingomyelinase/release of ceramide) loss of barrier function through ADAM10 activation	[34,35]
*Streptococcus pneumoniae*	pneumolysin	activation of Ca^2+^-dependent enzymes, including PKC-α activation of the NF-κB and p38 MAP kinase pathways	[36,37,38]
*Listeria monocytogenes*	listeriolysin O	dysfunction in the ENaC channel	[39]
Gram negative			
*Pseudomonas aeruginosa*	exoenzyme S and T	activation of TLR-2 and -4 disruption of the actin cytoskeleton and interference of phagocytosis	[40,41]
	exoenzyme Y and U	microtubule breakdown and tau phosphorylation	[42,43]
	LasB	cleavage of VE cadherin	[44]
*Bordetella pertussis*	pertussis toxin	increase in PKC-mediated endothelial permeability	[45]
*Shiga toxin such as Escherichia coli*	subtilase cytotoxin AB	inhibition of protein synthesis and induction of vacuole formation	[46,47]
	shigatoxin 2	increase in cytokine and chemokine expression, e.g., TNF-α, IL-6, IL-8 inhibition of protein synthesis and induction of ribotoxic and ER stress responses	[46,48]

A disintegrin and metalloproteinase domain-containing protein, ADAM10; epithelial sodium channel, ENaC; elastase B, LasB; mitogen-activated protein, MAP; vascular endothelial cadherin, VE cadherin.

**Table 2:** Influence of selected virus species on pulmonary endothelial function.

	Primary Site of Lung Cell Damage	Specific Impact on Pulmonary EC	Sources
Orthomyxoviridae			
*Influenza A*	EC and epithelial cells	increase in cytoplasmatic translocation of High-Mobility Group Box 1 (HMGB1); release of HMGB1 via IL-6-receptor and activation of Janus kinase signal transducer and activator of transcription 3 (JAK/STAT3) signaling pathway activation of p38 MAPK and c-Jun N -terminal kinase pathways leading to cytoskeletal rearrangement and hyperpermeability via e.g., ERM (ezrin, radixin and moesin) phosphorylation	[66,67,68]
Paramyxoviridae			
*RSV*	EC and epithelial cells	upregulation of intercellular adhesion molecule 1 (ICAM-1)/vascular cell adhesion molecule 1 (VCAM-1) and E-selectin upregulation (dependent on protein kinase C (PKC), protein kinase A (PKA), p38 MAPK) promotes PMN transmigration	[69]
*Human Metapneumovirus*	epithelial cells, alveolar macrophages and dendritic cells	indirect impact on EC via triggering thymic stromal lymphopoietin (TSLP), IL-8 and IL-33 expression in epithelial cells, cytokines IL-4, IL-5, Interferon γ (IFN-γ), IL-10, and TNF-α	[61,70,71]
Coronaviridae			
*SARS-CoV2*	ciliated bronchial cells, alveolar cells and EC	dysfunction of bradykinin–kallikrein pathway and RAAS complex by angiotensin-converting enzyme 2 (ACE2) downregulation via ADAM17 mediated ACE2 shedding decrease in platelet-derived growth factor receptor β (PDGFR-β) and Angiopoietin I through pericyte loss	[72,73,74]
*MERS-CoV*	ciliated bronchial cells, alveolar cells and EC	upregulation of proinflammatory cytokines e.g., TNF-α, IL-6, CSF-1 and CSF-3, IL-32 endoplasmic reticulum stress and oxysterols enhance apoptosis	[75]
Bunyaviridae			
*Hantavirus species*	epithelial and EC	induction of transcriptional activation of VEGF and expression of B cell lymphoma 2 (Bcl2) gene activation of NF-κB induction of the expression of the chemokines RANTES (regulated upon activation, normal T cell expressed and presumably secreted) and enhancement of IP-10 infiltration of CD4^+^ and CD8^+^ T cells	[76,77]

**Table 3:** Effects of several fungal pathogens on lung endothelial cells in invasive mycoses.

Fungal Species	Pathogens	Specific Impact on Pulmonary EC	Sources
*Candida albicans*	mannan, chitins, β-1,3-glucans, β-1,6-glucans	recognition by pattern recognition receptors (PRR), e.g., mannose receptors and TLR-2 and -4, presumably enablement of adhesion to and transmigration across EC	[95,96]
	secreted aspartic proteases (Sap2, Sap6)	potent induction of IL-1β, TNF-α, and IL-6 production, e.g., through activation of NLRP3 inflammasome	[97,98]
	candidalysin	formation of pores in host cell membrane, induction of potassium efflux to cause NLRP3 inflammasome activation	[99,100]
	Als3 (agglutinin-like sequence 3)	Induces tyrosine phosphorylation of EC proteins, causing microfilament rearrangement resulting in pseudopod production and endocytosis	[101]
*Aspergillus fumigatus*	galactosamino-galactan	inhibition of translation by ribosome immobilization, induction of endoplasmic reticulum stress and triggers NLRP3 inflammasome activation	[102]
	dsDNA	induction of AIM2 inflammasome	[103]
	thaumatin-like protein CalA	interaction with integrin α5β1 on EC, inducing endocytosis	[104]
	gliotoxin	inhibition of NF-κB pathway, anti-angiogenetic activity	[105]
*Rhizopus oryzae*	coat protein homolog (CotH2 and CotH3)	binding via glucose-regulated protein 78 to EC and induction of endocytosis	[106,107]

Absence in melanoma protein 2, AIM2; nucleotide-binding domain and leucine-rich repeat family pyrin domain-containing 3, NLRP3.

## Data Availability

Not applicable.

## Abbreviations

AA	arachidonic acid
ACE2	angiotensin-converting enzyme 2
ADAM	A disintegrin and metalloproteinase domain-containing protein
AIM2	absence in melanoma protein 2
ALI	acute lung injury
ARDS	Acute Respiratory Distress Syndrome
BMP-2	bone morphogenetic protein-2
CotH	coat protein homolog
COX	cyclooxygenase
COXIB	selective COX-2 inhibitor
CREB	cAMP response element-binding protein
CV-2	crossveinless-2
EC	endothelial cells
EGFR	epidermal growth factor receptor
ENaC	epithelial sodium channel
ERK-1/2	extracellular signal-regulated kinase 1/2
HDAC	histone deacetylase
HIF	hypoxia inducible factor
HMGB	high mobility group box
HMVEC	human microvascular endothelial cell
HPAEC	human pulmonary artery endothelial cells
HPMVEC	human pulmonary microvascular endothelial cell
HPS	hepatopulmonary syndrome
HPV	hypoxic pulmonary vasoconstriction
Hsp	heat shock protein
HUVEC	human umbilical vein endothelial cell
ICAM	intercellular adhesion molecule
IL	interleukin
IPA	invasive pulmonary aspergillosis
JAK/STAT3	Janus kinase signal transducer and activator of transcription
LasB	elastase B
LPS	lipopolysaccharide
LTA	lipoteichoic acid
MyD88	myeloid differentiation primary response 88
NFAT	nuclear factor of activated T cells
NF-κB	nuclear factor kappa B
NLRP3	nucleotide-binding domain and leucine-rich repeat family pyrin domain-containing 3
NK1R	neurokinin-1 receptor
Nrf2	nuclear factor erythroid 2-related factor 2
p38 MAPK	p38 mitogen-activated protein kinase
PAMP	pathogen-associated membrane pattern
PGD_2_	prostaglandin D_2_
PGE_2_	prostaglandin E_2_
PGF_2α_	prostaglandin F_2α_
PGG_2_	prostaglandin G_2_
PGH_2_	prostaglandin H_2_
PGI_2_	prostaglandin I_2_
PKA	protein kinase A
PKC	protein kinase C
Rac1	Ras-related C3 botulinum toxin substrate 1
Rap1	Ras-related protein 1
S1P	sphingosine-1-phosphate
TLR	toll-like receptor
TNF	tumor necrosis factor
TSLP	thymic stromal lymphopoietin
TXA2	thromboxane A_2_
VASP	vasodilator-stimulated phosphoprotein
VCAM	vascular cell adhesion molecule
VE cadherin	vascular endothelial cadherin
VEGF	vascular endothelial growth factor
VILI	ventilator induced lung injury
ZO-1	zona occludens- 1 –protein

## References

[1] Green C.E., Turner A.M. (2017). The role of the endothelium in asthma and chronic obstructive pulmonary disease (COPD). Respir. Res..

[2] Maniatis N.A., Kotanidou A., Catravas J.D., Orfanos S.E. (2008). Endothelial pathomechanisms in acute lung injury. Vasc. Pharmacol..

[3] Dejana E. (2004). Endothelial cell-cell junctions: Happy together. Nat. Rev. Mol. Cell Biol..

[4] Aird W.C. (2007). Phenotypic heterogeneity of the endothelium: II. Representative vascular beds. Circ. Res..

[5] Aird W.C. (2007). Phenotypic heterogeneity of the endothelium: I. Structure, function, and mechanisms. Circ. Res..

[6] Regan E.R., Aird W.C. (2012). Dynamical systems approach to endothelial heterogeneity. Circ. Res..

[7] Harb R., Whiteus C., Freitas C., Grutzendler J. (2013). In vivo imaging of cerebral microvascular plasticity from birth to death. J. Cereb. Blood Flow Metab..

[8] Gillich A., Zhang F., Farmer C.G., Travaglini K.J., Tan S.Y., Gu M., Zhou B., Feinstein J.A., Krasnow M.A., Metzger R.J. (2020). Capillary cell-type specialization in the alveolus. Nature.

[9] Troeger C., Blacker B., Khalil I.A., Rao P.C., Cao J., Zimsen S.R.M., Albertson S.B., Deshpande A., Farag T., Abebe Z. (2018). Estimates of the global, regional, and national morbidity, mortality, and aetiologies of lower respiratory infections in 195 countries, 1990–2016: A systematic analysis for the Global Burden of Disease Study 2016. Lancet Infect. Dis..

[10] Rubenfeld G.D., Caldwell E., Peabody E., Weaver J., Martin D.P., Neff M., Stern E.J., Hudson L.D. (2005). Incidence and outcomes of acute lung injury. N. Engl. J. Med..

[11] Catravas J.D., Snead C., Dimitropoulou C., Chang A.S.Y., Lucas R., Verin A.D., Black S.M. (2010). Harvesting, identification and barrier function of human lung microvascular endothelial cells. Vasc. Pharmacol..

[12] Gong P., Angelini D.J., Yang S., Xia G., Cross A.S., Mann D., Bannerman D.D., Vogel S.N., Goldblum S.E. (2008). TLR4 signaling is coupled to SRC family kinase activation, tyrosine phosphorylation of zonula adherens proteins, and opening of the paracellular pathway in human lung microvascular endothelia. J. Biol. Chem..

[13] Chatterjee A., Snead C., Yetik-Anacak G., Antonova G., Zeng J., Catravas J.D. (2008). Heat shock protein 90 inhibitors attenuate LPS-induced endothelial hyperpermeability. Am. J. Physiol. Lung Cell. Mol. Physiol..

[14] Jean-Baptiste E. (2007). Cellular mechanisms in sepsis. J. Intensive Care Med..

[15] Mehta D., Malik A.B. (2006). Signaling mechanisms regulating endothelial permeability. Physiol. Rev..

[16] Baumgarten G., Knuefermann P., Wrigge H., Putensen C., Stapel H., Fink K., Meyer R., Hoeft A., Grohé C. (2006). Role of Toll-like receptor 4 for the pathogenesis of acute lung injury in Gram-negative sepsis. Eur. J. Anaesthesiol..

[17] Park I., Kim M., Choe K., Song E., Seo H., Hwang Y., Ahn J., Lee S.-H., Lee J.H., Jo Y.H. (2019). Neutrophils disturb pulmonary microcirculation in sepsis-induced acute lung injury. Eur. Respir. J..

[18] Wiener-Kronish J.P., Albertine K.H., Matthay M.A. (1991). Differential responses of the endothelial and epithelial barriers of the lung in sheep to Escherichia coli endotoxin. J. Clin. Investig..

[19] Okajima K., Harada N. (2006). Regulation of inflammatory responses by sensory neurons: Molecular mechanism(s) and possible therapeutic applications. Curr. Med. Chem..

[20] Inagawa R., Okada H., Takemura G., Suzuki K., Takada C., Yano H., Ando Y., Usui T., Hotta Y., Miyazaki N. (2018). Ultrastructural Alteration of Pulmonary Capillary Endothelial Glycocalyx During Endotoxemia. Chest.

[21] Christaki E., Opal S.M. (2008). Is the mortality rate for septic shock really decreasing?. Curr. Opin. Crit. Care.

[22] Bannerman D.D., Goldblum S.E. (1997). Endotoxin induces endothelial barrier dysfunction through protein tyrosine phosphorylation. Am. J. Physiol..

[23] Barabutis N., Handa V., Dimitropoulou C., Rafikov R., Snead C., Kumar S., Joshi A., Thangjam G., Fulton D., Black S.M. (2013). LPS induces pp60c-src-mediated tyrosine phosphorylation of Hsp90 in lung vascular endothelial cells and mouse lung. Am. J. Physiol. Lung Cell. Mol. Physiol..

[24] Joshi A.D., Dimitropoulou C., Thangjam G., Snead C., Feldman S., Barabutis N., Fulton D., Hou Y., Kumar S., Patel V. (2014). Heat shock protein 90 inhibitors prevent LPS-induced endothelial barrier dysfunction by disrupting RhoA signaling. Am. J. Respir. Cell Mol. Biol..

[25] Kása A., Csortos C., Verin A.D. (2015). Cytoskeletal mechanisms regulating vascular endothelial barrier function in response to acute lung injury. Tissue Barriers.

[26] Kovacs-Kasa A., Gorshkov B.A., Kim K.-M., Kumar S., Black S.M., Fulton D.J., Dimitropoulou C., Catravas J.D., Verin A.D. (2016). The protective role of MLCP-mediated ERM dephosphorylation in endotoxin-induced lung injury in vitro and in vivo. Sci. Rep..

[27] Kovacs-Kasa A., Kim K.M., Cherian-Shaw M., Black S.M., Fulton D.J., Verin A.D. (2018). Extracellular adenosine-induced Rac1 activation in pulmonary endothelium: Molecular mechanisms and barrier-protective role. J. Cell. Physiol..

[28] Kuhlmann N., Wroblowski S., Knyphausen P., Boor S.d., Brenig J., Zienert A.Y., Meyer-Teschendorf K., Praefcke G.J.K., Nolte H., Krüger M. (2016). Structural and Mechanistic Insights into the Regulation of the Fundamental Rho Regulator RhoGDIα by Lysine Acetylation. J. Biol. Chem..

[29] Fontaine S.N., Sabbagh J.J., Baker J., Martinez-Licha C.R., Darling A., Dickey C.A. (2015). Cellular factors modulating the mechanism of tau protein aggregation. Cell. Mol. Life Sci..

[30] Kovacs-Kasa A., Kovacs L., Cherian-Shaw M., Patel V., Meadows M.L., Fulton D.J., Su Y., Verin A.D. (2021). Inhibition of Class IIa HDACs improves endothelial barrier function in endotoxin-induced acute lung injury. J. Cell. Physiol..

[31] Takeda K., Akira S. (2005). Toll-like receptors in innate immunity. Int. Immunol..

[32] Pietrocola G., Arciola C.R., Rindi S., Di Poto A., Missineo A., Montanaro L., Speziale P. (2011). Toll-like receptors (TLRs) in innate immune defense against Staphylococcus aureus. Int. J. Artif. Organs.

[33] Pai A.B., Patel H., Prokopienko A.J., Alsaffar H., Gertzberg N., Neumann P., Punjabi A., Johnson A. (2012). Lipoteichoic acid from Staphylococcus aureus induces lung endothelial cell barrier dysfunction: Role of reactive oxygen and nitrogen species. PLoS ONE.

[34] Czajkowsky D.M., Sheng S., Shao Z. (1998). Staphylococcal alpha-hemolysin can form hexamers in phospholipid bilayers. J. Mol. Biol..

[35] Becker K.A., Fahsel B., Kemper H., Mayeres J., Li C., Wilker B., Keitsch S., Soddemann M., Sehl C., Kohnen M. (2018). Staphylococcus aureus Alpha-Toxin Disrupts Endothelial-Cell Tight Junctions via Acid Sphingomyelinase and Ceramide. Infect. Immun..

[36] Lucas R., Yang G., Gorshkov B.A., Zemskov E.A., Sridhar S., Umapathy N.S., Jezierska-Drutel A., Alieva I.B., Leustik M., Hossain H. (2012). Protein kinase C-α and arginase I mediate pneumolysin-induced pulmonary endothelial hyperpermeability. Am. J. Respir. Cell Mol. Biol..

[37] Zhou A., Wang H., Lan K., Zhang X., Xu W., Yin Y., Li D., Yuan J., He Y. (2012). Apoptosis induced by pneumolysin in human endothelial cells involves mitogen-activated protein kinase phosphorylation. Int. J. Mol. Med..

[38] N’Guessan P.D., Schmeck B., Ayim A., Hocke A.C., Brell B., Hammerschmidt S., Rosseau S., Suttorp N., Hippenstiel S. (2005). Streptococcus pneumoniae R6x induced p38 MAPK and JNK-mediated caspase-dependent apoptosis in human endothelial cells. Thromb. Haemost..

[39] Czikora I., Alli A.A., Sridhar S., Matthay M.A., Pillich H., Hudel M., Berisha B., Gorshkov B., Romero M.J., Gonzales J. (2017). Epithelial Sodium Channel-α Mediates the Protective Effect of the TNF-Derived TIP Peptide in Pneumolysin-Induced Endothelial Barrier Dysfunction. Front. Immunol..

[40] Epelman S., Stack D., Bell C., Wong E., Neely G.G., Krutzik S., Miyake K., Kubes P., Zbytnuik L.D., Ma L.L. (2004). Different domains of Pseudomonas aeruginosa exoenzyme S activate distinct TLRs. J. Immunol..

[41] Huber P., Bouillot S., Elsen S., Attrée I. (2014). Sequential inactivation of Rho GTPases and Lim kinase by Pseudomonas aeruginosa toxins ExoS and ExoT leads to endothelial monolayer breakdown. Cell. Mol. Life Sci..

[42] Sayner S.L., Frank D.W., King J., Chen H., VandeWaa J., Stevens T. (2004). Paradoxical cAMP-induced lung endothelial hyperpermeability revealed by Pseudomonas aeruginosa ExoY. Circ. Res..

[43] Ochoa C.D., Alexeyev M., Pastukh V., Balczon R., Stevens T. (2012). Pseudomonas aeruginosa exotoxin Y is a promiscuous cyclase that increases endothelial tau phosphorylation and permeability. J. Biol. Chem..

[44] Golovkine G., Faudry E., Bouillot S., Voulhoux R., Attrée I., Huber P. (2014). VE-cadherin cleavage by LasB protease from Pseudomonas aeruginosa facilitates type III secretion system toxicity in endothelial cells. PLoS Pathog..

[45] Tsan M.F., Cao X., White J.E., Sacco J., Lee C.Y. (1999). Pertussis toxin-induced lung edema. Role of manganese superoxide dismutase and protein kinase C. Am. J. Respir. Cell Mol. Biol..

[46] Wang H., Rogers T.J., Paton J.C., Paton A.W. (2014). Differential effects of Escherichia coli subtilase cytotoxin and Shiga toxin 2 on chemokine and proinflammatory cytokine expression in human macrophage, colonic epithelial, and brain microvascular endothelial cell lines. Infect. Immun..

[47] Morinaga N., Yahiro K., Matsuura G., Watanabe M., Nomura F., Moss J., Noda M. (2007). Two distinct cytotoxic activities of subtilase cytotoxin produced by shiga-toxigenic Escherichia coli. Infect. Immun..

[48] Lee M.S., Kim M.H., Tesh V.L. (2013). Shiga toxins expressed by human pathogenic bacteria induce immune responses in host cells. J. Microbiol..

[49] Wong K.T., Shieh W.-J., Kumar S., Norain K., Abdullah W., Guarner J., Goldsmith C.S., Chua K.B., Lam S.K., Tan C.T. (2002). Nipah Virus Infection. Am. J. Pathol..

[50] Ackermann M., Verleden S.E., Kuehnel M., Haverich A., Welte T., Laenger F., Vanstapel A., Werlein C., Stark H., Tzankov A. (2020). Pulmonary Vascular Endothelialitis, Thrombosis, and Angiogenesis in Covid-19. N. Engl. J. Med..

[51] Geisbert T.W., Daddario-DiCaprio K.M., Hickey A.C., Smith M.A., Chan Y.-P., Wang L.-F., Mattapallil J.J., Geisbert J.B., Bossart K.N., Broder C.C. (2010). Development of an acute and highly pathogenic nonhuman primate model of Nipah virus infection. PLoS ONE.

[52] Jessie K., Fong M.Y., Devi S., Lam S.K., Wong K.T. (2004). Localization of dengue virus in naturally infected human tissues, by immunohistochemistry and in situ hybridization. J. Infect. Dis..

[53] Zaki S.R., Greer P.W., Coffield L.M., Goldsmith C.S., Nolte K.B., Foucar K., Feddersen R.M., Zumwalt R.E., Miller G.L., Khan A.S. (1995). Hantavirus pulmonary syndrome. Pathogenesis of an emerging infectious disease. Am. J. Pathol..

[54] Olofsson S., Bergström T. (2005). Glycoconjugate glycans as viral receptors. Ann. Med..

[55] Seternes T., Sørensen K., Smedsrød B. (2002). Scavenger endothelial cells of vertebrates: A nonperipheral leukocyte system for high-capacity elimination of waste macromolecules. Proc. Natl. Acad. Sci. USA.

[56] Teijaro J.R., Walsh K.B., Cahalan S., Fremgen D.M., Roberts E., Scott F., Martinborough E., Peach R., Oldstone M.B.A., Rosen H. (2011). Endothelial cells are central orchestrators of cytokine amplification during influenza virus infection. Cell.

[57] Peiris J.S.M., Cheung C.Y., Leung C.Y.H., Nicholls J.M. (2009). Innate immune responses to influenza A H5N1: Friend or foe?. Trends Immunol..

[58] Varga Z., Flammer A.J., Steiger P., Haberecker M., Andermatt R., Zinkernagel A.S., Mehra M.R., Schuepbach R.A., Ruschitzka F., Moch H. (2020). Endothelial cell infection and endotheliitis in COVID-19. Lancet.

[59] Puerta-Guardo H., Glasner D.R., Harris E. (2016). Dengue Virus NS1 Disrupts the Endothelial Glycocalyx, Leading to Hyperpermeability. PLoS Pathog..

[60] Gorbunova E.E., Simons M.J., Gavrilovskaya I.N., Mackow E.R. (2016). The Andes Virus Nucleocapsid Protein Directs Basal Endothelial Cell Permeability by Activating RhoA. mBio.

[61] Kolli D., Gupta M.R., Sbrana E., Velayutham T.S., Chao H., Casola A., Garofalo R.P. (2014). Alveolar macrophages contribute to the pathogenesis of human metapneumovirus infection while protecting against respiratory syncytial virus infection. Am. J. Respir. Cell Mol. Biol..

[62] Haeberle H.A., Takizawa R., Casola A., Brasier A.R., Dieterich H.J., Van Rooijen N., Gatalica Z., Garofalo R.P. (2002). Respiratory syncytial virus-induced activation of nuclear factor-kappaB in the lung involves alveolar macrophages and toll-like receptor 4-dependent pathways. J. Infect. Dis..

[63] Morris D.E., Cleary D.W., Clarke S.C. (2017). Secondary Bacterial Infections Associated with Influenza Pandemics. Front. Microbiol..

[64] Shafran N., Shafran I., Ben-Zvi H., Sofer S., Sheena L., Krause I., Shlomai A., Goldberg E., Sklan E.H. (2021). Secondary bacterial infection in COVID-19 patients is a stronger predictor for death compared to influenza patients. Sci. Rep..

[65] Koch R.M., Diavatopoulos D.A., Ferwerda G., Pickkers P., Jonge M.I.d., Kox M. (2018). The endotoxin-induced pulmonary inflammatory response is enhanced during the acute phase of influenza infection. Intensive Care Med. Exp..

[66] Ru Y.X., Li Y.C., Zhao Y., Zhao S.X., Yang J.P., Zhang H.M., Pang T.X. (2011). Multiple organ invasion by viruses: Pathological characteristics in three fatal cases of the 2009 pandemic influenza A/H1N1. Ultrastruct. Pathol..

[67] Nakajima N., Van Tin N., Sato Y., Thach H.N., Katano H., Diep P.H., Kumasaka T., Thuy N.T., Hasegawa H., San L.T. (2013). Pathological study of archival lung tissues from five fatal cases of avian H5N1 influenza in Vietnam. Mod. Pathol..

[68] Chatterjee A., Mavunda K., Krilov L.R. (2021). Current State of Respiratory Syncytial Virus Disease and Management. Infect. Dis. Ther..

[69] Arnold R., Konig W. (2005). Respiratory syncytial virus infection of human lung endothelial cells enhances selectively intercellular adhesion molecule-1 expression. J. Immunol..

[70] Lay M.K., Cespedes P.F., Palavecino C.E., Leon M.A., Diaz R.A., Salazar F.J., Mendez G.P., Bueno S.M., Kalergis A.M. (2015). Human metapneumovirus infection activates the TSLP pathway that drives excessive pulmonary inflammation and viral replication in mice. Eur. J. Immunol..

[71] Bugatti A., Marsico S., Fogli M., Roversi S., Messali S., Bosisio D., Giagulli C., Caruso A., Sozzani S., Fiorentini S. (2020). Human Metapneumovirus Establishes Persistent Infection in Lung Microvascular Endothelial Cells and Primes a Th2-Skewed Immune Response. Microorganisms.

[72] Clausen T.M., Sandoval D.R., Spliid C.B., Pihl J., Perrett H.R., Painter C.D., Narayanan A., Majowicz S.A., Kwong E.M., McVicar R.N. (2020). SARS-CoV-2 Infection Depends on Cellular Heparan Sulfate and ACE2. Cell.

[73] He L., Mäe M.A., Muhl L., Sun Y., Pietilä R., Nahar K., Liébanas E.V., Fagerlund M.J., Oldner A., Liu J. (2020). Pericyte-specific vascular expression of SARS-CoV-2 receptor ACE2—Implications for microvascular inflammation and hypercoagulopathy in COVID-19. bioRxiv.

[74] Wan Y., Shang J., Graham R., Baric R.S., Li F., Gallagher T. (2020). Receptor Recognition by the Novel Coronavirus from Wuhan: An Analysis Based on Decade-Long Structural Studies of SARS Coronavirus. J. Virol..

[75] Widagdo W., Sooksawasdi Na Ayudhya S., Hundie G.B., Haagmans B.L. (2019). Host Determinants of MERS-CoV Transmission and Pathogenesis. Viruses.

[76] Geimonen E., Neff S., Raymond T., Kocer S.S., Gavrilovskaya I.N., Mackow E.R. (2002). Pathogenic and nonpathogenic hantaviruses differentially regulate endothelial cell responses. Proc. Natl. Acad. Sci. USA.

[77] Mori M., Rothman A.L., Kurane I., Montoya J.M., Nolte K.B., Norman J.E., Waite D.C., Koster F.T., Ennis F.A. (1999). High levels of cytokine-producing cells in the lung tissues of patients with fatal hantavirus pulmonary syndrome. J. Infect. Dis..

[78] Patterson T.F., Kirkpatrick W.R., White M., Hiemenz J.W., Wingard J.R., Dupont B., Rinaldi M.G., Stevens D.A., Graybill J.R. (2000). Invasive aspergillosis. Disease spectrum, treatment practices, and outcomes. I3 Aspergillus Study Group. Medicine.

[79] Upton A., Kirby K.A., Carpenter P., Boeckh M., Marr K.A. (2007). Invasive aspergillosis following hematopoietic cell transplantation: Outcomes and prognostic factors associated with mortality. Clin. Infect. Dis..

[80] Al-Bader N., Vanier G., Liu H., Gravelat F.N., Urb M., Hoareau C.M.-Q., Campoli P., Chabot J., Filler S.G., Sheppard D.C. (2010). Role of trehalose biosynthesis in Aspergillus fumigatus development, stress response, and virulence. Infect. Immun..

[81] Ejzykowicz D.E., Cunha M.M., Rozental S., Solis N.V., Gravelat F.N., Sheppard D.C., Filler S.G. (2009). The Aspergillus fumigatus transcription factor Ace2 governs pigment production, conidiation and virulence. Mol. Microbiol..

[82] Ejzykowicz D.E., Solis N.V., Gravelat F.N., Chabot J., Li X., Sheppard D.C., Filler S.G. (2010). Role of Aspergillus fumigatus DvrA in host cell interactions and virulence. Eukaryot. Cell.

[83] Van de Veerdonk F.L., Joosten L.A.B., Devesa I., Mora-Montes H.M., Kanneganti T.-D., Dinarello C.A., van der Meer J.W.M., Gow N.A.R., Kullberg B.J., Netea M.G. (2009). Bypassing pathogen-induced inflammasome activation for the regulation of interleukin-1beta production by the fungal pathogen Candida albicans. J. Infect. Dis..

[84] Ibrahim A.S., Spellberg B., Avanessian V., Fu Y., Edwards J.E. (2005). Rhizopus oryzae adheres to, is phagocytosed by, and damages endothelial cells in vitro. Infect. Immun..

[85] Chiang L.Y., Sheppard D.C., Gravelat F.N., Patterson T.F., Filler S.G. (2008). Aspergillus fumigatus stimulates leukocyte adhesion molecules and cytokine production by endothelial cells in vitro and during invasive pulmonary disease. Infect. Immun..

[86] Phan Q.T., Belanger P.H., Filler S.G. (2000). Role of hyphal formation in interactions of Candida albicans with endothelial cells. Infect. Immun..

[87] Lopes Bezerra L.M., Filler S.G. (2004). Interactions of Aspergillus fumigatus with endothelial cells: Internalization, injury, and stimulation of tissue factor activity. Blood.

[88] Frater J.L., Hall G.S., Procop G.W. (2001). Histologic features of zygomycosis: Emphasis on perineural invasion and fungal morphology. Arch. Pathol. Lab. Med..

[89] Shaukat A., Bakri F., Young P., Hahn T., Ball D., Baer M.R., Wetzler M., Slack J.L., Loud P., Czuczman M. (2005). Invasive filamentous fungal infections in allogeneic hematopoietic stem cell transplant recipients after recovery from neutropenia: Clinical, radiologic, and pathologic characteristics. Mycopathologia.

[90] Kamai Y., Lossinsky A.S., Liu H., Sheppard D.C., Filler S.G. (2009). Polarized response of endothelial cells to invasion by Aspergillus fumigatus. Cell. Microbiol..

[91] Banoth B., Tuladhar S., Karki R., Sharma B.R., Briard B., Kesavardhana S., Burton A., Kanneganti T.-D. (2020). ZBP1 promotes fungi-induced inflammasome activation and pyroptosis, apoptosis, and necroptosis (PANoptosis). J. Biol. Chem..

[92] Stergiopoulou T., Meletiadis J., Roilides E., Kleiner D.E., Schaufele R., Roden M., Harrington S., Dad L., Segal B., Walsh T.J. (2007). Host-dependent patterns of tissue injury in invasive pulmonary aspergillosis. Am. J. Clin. Pathol..

[93] Coelho C., Bocca A.L., Casadevall A. (2014). The tools for virulence of Cryptococcus neoformans. Adv. Appl. Microbiol..

[94] Gilbert A.S., Wheeler R.T., May R.C. (2014). Fungal Pathogens: Survival and Replication within Macrophages. Cold Spring Harb. Perspect. Med..

[95] Opitz B., Hippenstiel S., Eitel J., Suttorp N. (2007). Extra- and intracellular innate immune recognition in endothelial cells. Thromb. Haemost..

[96] Netea M.G., van der Graaf C.A.A., Vonk A.G., Verschueren I., van der Meer J.W.M., Kullberg B.J. (2002). The role of toll-like receptor (TLR) 2 and TLR4 in the host defense against disseminated candidiasis. J. Infect. Dis..

[97] Pietrella D., Pandey N., Gabrielli E., Pericolini E., Perito S., Kasper L., Bistoni F., Cassone A., Hube B., Vecchiarelli A. (2013). Secreted aspartic proteases of Candida albicans activate the NLRP3 inflammasome. Eur. J. Immunol..

[98] Pietrella D., Rachini A., Pandey N., Schild L., Netea M., Bistoni F., Hube B., Vecchiarelli A. (2010). The Inflammatory response induced by aspartic proteases of Candida albicans is independent of proteolytic activity. Infect. Immun..

[99] Kasper L., König A., Koenig P.-A., Gresnigt M.S., Westman J., Drummond R.A., Lionakis M.S., Groß O., Ruland J., Naglik J.R. (2018). The fungal peptide toxin Candidalysin activates the NLRP3 inflammasome and causes cytolysis in mononuclear phagocytes. Nat. Commun..

[100] Naglik J.R., Gaffen S.L., Hube B. (2019). Candidalysin: Discovery and function in Candida albicans infections. Curr. Opin. Microbiol..

[101] Phan Q.T., Myers C.L., Fu Y., Sheppard D.C., Yeaman M.R., Welch W.H., Ibrahim A.S., Edwards J.E., Filler S.G. (2007). Als3 is a Candida albicans invasin that binds to cadherins and induces endocytosis by host cells. PLoS Biol..

[102] Briard B., Fontaine T., Samir P., Place D.E., Muszkieta L., Malireddi R.K.S., Karki R., Christgen S., Bomme P., Vogel P. (2020). Galactosaminogalactan activates the inflammasome to provide host protection. Nature.

[103] Briard B., Malireddi R.K.S., Kanneganti T.-D. (2021). Role of inflammasomes/pyroptosis and PANoptosis during fungal infection. PLoS Pathog..

[104] Liu H., Lee M.J., Solis N.V., Phan Q.T., Swidergall M., Ralph B., Ibrahim A.S., Sheppard D.C., Filler S.G. (2016). *Aspergillus fumigatus* CalA binds to integrin α5β1 and mediates host cell invasion. Nat. Microbiol..

[105] Lee H.J., Lee J.H., Hwang B.Y., Kim H.S., Lee J.J. (2001). Anti-angiogenic activities of gliotoxin and its methylthioderivative, fungal metabolites. Arch. Pharm. Res..

[106] Liu M., Spellberg B., Phan Q.T., Fu Y., Fu Y., Lee A.S., Edwards J.E., Filler S.G., Ibrahim A.S. (2010). The endothelial cell receptor GRP78 is required for mucormycosis pathogenesis in diabetic mice. J. Clin. Investig..

[107] Gebremariam T., Liu M., Luo G., Bruno V., Phan Q.T., Waring A.J., Edwards J.E., Filler S.G., Yeaman M.R., Ibrahim A.S. (2014). CotH3 mediates fungal invasion of host cells during mucormycosis. J. Clin. Investig..

[108] Tremblay L.N., Slutsky A.S. (2006). Ventilator-induced lung injury: From the bench to the bedside. Intensive Care Med..

[109] Birukov K.G., Jacobson J.R., Flores A.A., Ye S.Q., Birukova A.A., Verin A.D., Garcia J.G.N. (2003). Magnitude-dependent regulation of pulmonary endothelial cell barrier function by cyclic stretch. Am. J. Physiol. Lung Cell. Mol. Physiol..

[110] Birukova A.A., Rios A., Birukov K.G. (2008). Long-term cyclic stretch controls pulmonary endothelial permeability at translational and post-translational levels. Exp. Cell Res..

[111] Wolfson R.K., Mapes B., Garcia J.G.N. (2014). Excessive mechanical stress increases HMGB1 expression in human lung microvascular endothelial cells via STAT3. Microvasc. Res..

[112] Wolfson R.K., Chiang E.T., Garcia J.G.N. (2011). HMGB1 induces human lung endothelial cell cytoskeletal rearrangement and barrier disruption. Microvasc. Res..

[113] Vestweber D., Winderlich M., Cagna G., Nottebaum A.F. (2009). Cell adhesion dynamics at endothelial junctions: VE-cadherin as a major player. Trends Cell Biol..

[114] Dejana E., Lampugnani M.G., Martinez-Estrada O., Bazzoni G. (2000). The molecular organization of endothelial junctions and their functional role in vascular morphogenesis and permeability. Int. J. Dev. Biol..

[115] Schulte D., Küppers V., Dartsch N., Broermann A., Li H., Zarbock A., Kamenyeva O., Kiefer F., Khandoga A., Massberg S. (2011). Stabilizing the VE-cadherin-catenin complex blocks leukocyte extravasation and vascular permeability. EMBO J..

[116] Ware L.B., Matthay M.A. (2000). The acute respiratory distress syndrome. N. Engl. J. Med..

[117] Matthay M.A., Zimmerman G.A. (2005). Acute lung injury and the acute respiratory distress syndrome: Four decades of inquiry into pathogenesis and rational management. Am. J. Respir. Cell Mol. Biol..

[118] Liu Y.-Y., Chiang C.-H., Chuang C.-H., Liu S.-L., Jheng Y.-H., Ryu J.H. (2014). Spillover of cytokines and reactive oxygen species in ventilator-induced lung injury associated with inflammation and apoptosis in distal organs. Respir. Care.

[119] Ma H., Feng X., Ding S. (2015). Hesperetin attenuates ventilator-induced acute lung injury through inhibition of NF-κB-mediated inflammation. Eur. J. Pharmacol..

[120] Iwaki M., Ito S., Morioka M., Iwata S., Numaguchi Y., Ishii M., Kondo M., Kume H., Naruse K., Sokabe M. (2009). Mechanical stretch enhances IL-8 production in pulmonary microvascular endothelial cells. Biochem. Biophys. Res. Commun..

[121] Miyao N., Suzuki Y., Takeshita K., Kudo H., Ishii M., Hiraoka R., Nishio K., Tamatani T., Sakamoto S., Suematsu M. (2006). Various adhesion molecules impair microvascular leukocyte kinetics in ventilator-induced lung injury. Am. J. Physiol. Lung Cell. Mol. Physiol..

[122] Lu Q., Zemskov E.A., Sun X., Wang H., Yegambaram M., Wu X., Garcia-Flores A., Song S., Tang H., Kangath A. (2021). Activation of the mechanosensitive Ca^2+^ channel TRPV4 induces endothelial barrier permeability via the disruption of mitochondrial bioenergetics. Redox Biol..

[123] Su K., Bo L., Jiang C., Deng X., Zhao Y.-Y., Minshall R.D., Hu G. (2021). TLR4 is required for macrophage efferocytosis during resolution of ventilator-induced lung injury. Am. J. Physiol. Lung Cell. Mol. Physiol..

[124] Determann R.M., Royakkers A., Wolthuis E.K., Vlaar A.P., Choi G., Paulus F., Hofstra J.J., de Graaff M.J., Korevaar J.C., Schultz M.J. (2010). Ventilation with lower tidal volumes as compared with conventional tidal volumes for patients without acute lung injury: A preventive randomized controlled trial. Crit. Care.

[125] Garcia J.G., Liu F., Verin A.D., Birukova A., Dechert M.A., Gerthoffer W.T., Bamberg J.R., English D. (2001). Sphingosine 1-phosphate promotes endothelial cell barrier integrity by Edg-dependent cytoskeletal rearrangement. J. Clin. Investig..

[126] Obinata H., Hla T. (2012). Sphingosine 1-phosphate in coagulation and inflammation. Semin. Immunopathol..

[127] Shea B.S., Brooks S.F., Fontaine B.A., Chun J., Luster A.D., Tager A.M. (2010). Prolonged exposure to sphingosine 1-phosphate receptor-1 agonists exacerbates vascular leak, fibrosis, and mortality after lung injury. Am. J. Respir. Cell Mol. Biol..

[128] London N.R., Zhu W., Bozza F.A., Smith M.C.P., Greif D.M., Sorensen L.K., Chen L., Kaminoh Y., Chan A.C., Passi S.F. (2010). Targeting Robo4-dependent Slit signaling to survive the cytokine storm in sepsis and influenza. Sci. Transl. Med..

[129] Jones C.A., Nishiya N., London N.R., Zhu W., Sorensen L.K., Chan A.C., Lim C.J., Chen H., Zhang Q., Schultz P.G. (2009). Slit2-Robo4 signalling promotes vascular stability by blocking Arf6 activity. Nat. Cell Biol..

[130] Wolfson R.K., Lang G., Jacobson J., Garcia J.G.N., Voelkel N.F., Rounds S. (2009). Therapeutic Strategies to Limit Lung Endothelial Cell Permeability. The Pulmonary Endothelium.

[131] Zarbock A., Singbartl K., Ley K. (2006). Complete reversal of acid-induced acute lung injury by blocking of platelet-neutrophil aggregation. J. Clin. Investig..

[132] Knight P.R., Druskovich G., Tait A.R., Johnson K.J. (1992). The role of neutrophils, oxidants, and proteases in the pathogenesis of acid pulmonary injury. Anesthesiology.

[133] Davidson B.A., Knight P.R., Helinski J.D., Nader N.D., Shanley T.P., Johnson K.J. (1999). The role of tumor necrosis factor-alpha in the pathogenesis of aspiration pneumonitis in rats. Anesthesiology.

[134] Folkesson H.G., Matthay M.A., Hébert C.A., Broaddus V.C. (1995). Acid aspiration-induced lung injury in rabbits is mediated by interleukin-8-dependent mechanisms. J. Clin. Investig..

[135] Knight P.R., Rutter T., Tait A.R., Coleman E., Johnson K. (1993). Pathogenesis of gastric particulate lung injury: A comparison and interaction with acidic pneumonitis. Anesth. Analg..

[136] Raghavendran K., Davidson B.A., Mullan B.A., Hutson A.D., Russo T.A., Manderscheid P.A., Woytash J.A., Holm B.A., Notter R.H., Knight P.R. (2005). Acid and particulate-induced aspiration lung injury in mice: Importance of MCP-1. Am. J. Physiol. Lung Cell. Mol. Physiol..

[137] Nawa S., Shimizu A., Kino K., Soga H., Shimizu N. (1994). Development of an experimental model of an acute respiratory failure by intratracheal sea water infusion: A comparison with a conventional oleic acid induction. Res. Exp. Med..

[138] Xinmin D., Yunyou D., Chaosheng P., Huasong F., Pingkun Z., Jiguang M., Zhiqian X., Qinzhi X. (2006). Dexamethasone treatment attenuates early seawater instillation-induced acute lung injury in rabbits. Pharmacol. Res..

[139] Li P.-C., Wang B.-R., Li C.-C., Lu X., Qian W.-S., Li Y.-J., Jin F.-G., Mu D.-G. (2018). Seawater inhalation induces acute lung injury via ROS generation and the endoplasmic reticulum stress pathway. Int. J. Mol. Med..

[140] Zhang M., Wang L., Dong M., Li Z., Jin F. (2014). Endothelial Semaphorin 7A promotes inflammation in seawater aspiration-induced acute lung injury. Int. J. Mol. Sci..

[141] Li J., Xu M., Fan Q., Xie X., Zhang Y., Mu D., Zhao P., Zhang B., Cao F., Wang Y. (2011). Tanshinone IIA ameliorates seawater exposure-induced lung injury by inhibiting aquaporins (AQP) 1 and AQP5 expression in lung. Respir. Physiol. Neurobiol..

[142] Williams C.S., Mann M., DuBois R.N. (1999). The role of cyclooxygenases in inflammation, cancer, and development. Oncogene.

[143] Rajakariar R., Yaqoob M.M., Gilroy D.W. (2006). COX-2 in inflammation and resolution. Mol. Interv..

[144] Jacobs E.R., Zeldin D.C. (2001). The lung HETEs (and EETs) up. Am. J. Physiol. Heart Circ. Physiol..

[145] Oni-Orisan A., Deng Y., Schuck R.N., Theken K.N., Edin M.L., Lih F.B., Molnar K., DeGraff L., Tomer K.B., Zeldin D.C. (2013). Dual modulation of cyclooxygenase and CYP epoxygenase metabolism and acute vascular inflammation in mice. Prostaglandins Other Lipid Mediat..

[146] Kis B., Snipes J.A., Busija D.W. (2005). Acetaminophen and the cyclooxygenase-3 puzzle: Sorting out facts, fictions, and uncertainties. J. Pharmacol. Exp. Ther..

[147] Mitchell J.A., Kirkby N.S., Ahmetaj-Shala B., Armstrong P.C., Crescente M., Ferreira P., Pires M.E.L., Vaja R., Warner T.D. (2021). Cyclooxygenases and the cardiovascular system. Pharmacol. Ther..

[148] Straus D.S., Glass C.K. (2001). Cyclopentenone prostaglandins: New insights on biological activities and cellular targets. Med. Res. Rev..

[149] Mbonye U.R., Song I. (2009). Posttranscriptional and posttranslational determinants of cyclooxygenase expression. BMB Rep..

[150] Smyth E.M., Grosser T., Wang M., Yu Y., FitzGerald G.A. (2009). Prostanoids in health and disease. J. Lipid Res..

[151] Gao Y., Zhang H., Luo L., Lin J., Li D., Zheng S., Huang H., Yan S., Yang J., Hao Y. (2017). Resolvin D1 Improves the Resolution of Inflammation via Activating NF-kappaB p50/p50-Mediated Cyclooxygenase-2 Expression in Acute Respiratory Distress Syndrome. J. Immunol..

[152] Hirata T., Narumiya S. (2011). Prostanoid receptors. Chem. Rev..

[153] Breyer R.M., Bagdassarian C.K., Myers S.A., Breyer M.D. (2001). Prostanoid Receptors: Subtypes and Signaling. Annu. Rev. Pharmacol. Toxicol..

[154] Narumiya S., Sugimoto Y., Ushikubi F. (1999). Prostanoid Receptors: Structures, Properties, and Functions. Physiol. Rev..

[155] Bos C.L., Richel D.J., Ritsema T., Peppelenbosch M.P., Versteeg H.H. (2004). Prostanoids and prostanoid receptors in signal transduction. Int. J. Biochem. Cell Biol..

[156] Alfranca A., Iñiguez M.A., Fresno M., Redondo J.M. (2006). Prostanoid signal transduction and gene expression in the endothelium: Role in cardiovascular diseases. Cardiovasc. Res..

[157] Ashton A.W., Cheng Y., Helisch A., Ware J.A. (2004). Thromboxane A2 receptor agonists antagonize the proangiogenic effects of fibroblast growth factor-2: Role of receptor internalization, thrombospondin-1, and αvβ3. Circ. Res..

[158] Dormond O., Bezzi M., Mariotti A., Ruëgg C. (2002). Prostaglandin E2 promotes integrin αVβ3-dependent endothelial cell adhesion, Rac-activation, and spreading through cAMP/PKA-dependent signaling. J. Biol. Chem..

[159] Bhattacharya M., Peri K., Ribeiro-da-Silva A., Almazan G., Shichi H., Hou X., Varma D.R., Chemtob S. (1999). Localization of functional prostaglandin E2 receptors EP3 and EP4 in the nuclear envelope. J. Biol. Chem..

[160] Kömhoff M., Lesener B., Nakao K., Seyberth H.W., Nüsing R.M. (1998). Localization of the prostacyclin receptor in human kidney. Kidney Int..

[161] Murata T., Lin M.I., Aritake K., Matsumoto S., Narumiya S., Ozaki H., Urade Y., Hori M., Sessa W.C. (2008). Role of prostaglandin D_2_ receptor DP as a suppressor of tumor hyperpermeability and angiogenesis in vivo. Proc. Natl. Acad. Sci. USA.

[162] Chen J., Champa-Rodriguez M., Woodward D. (1995). Identification of a prostanoid FP receptor population producing endothelium-dependent vasorelaxation in the rabbit jugular vein. Br. J. Pharmacol..

[163] Oskolkova O., Gawlak G., Tian Y.F., Ke Y.B., Sarich N., Son S., Andreasson K., Bochkov V.N., Birukova A.A., Birukov K.G. (2017). Prostaglandin E receptor-4 receptor mediates endothelial barrier-enhancing and anti-inflammatory effects of oxidized phospholipids. FASEB J..

[164] Starosta V., Wu T., Zimman A., Pham D., Tian X., Oskolkova O., Bochkov V., Berliner J.A., Birukova A.A., Birukov K.G. (2012). Differential regulation of endothelial cell permeability by high and low doses of oxidized 1-palmitoyl-2-arachidonyl-sn-glycero-3-phosphocholine. Am. J. Respir. Cell Mol. Biol..

[165] Göggel R., Hoffman S., Nüsing R., Narumiya S., Uhlig S. (2002). Platelet-Activating Factor–induced Pulmonary Edema Is Partly Mediated by Prostaglandin E2, E-Prostanoid 3-Receptors, and Potassium Channels. Am. J. Respir. Crit. Care Med..

[166] Rittchen S., Rohrer K., Platzer W., Knuplez E., Bärnthaler T., Marsh L.M., Atallah R., Sinn K., Klepetko W., Sharma N. (2020). Prostaglandin D_2_ strengthens human endothelial barrier by activation of E-type receptor 4. Biochem. Pharmacol..

[167] Ke Y., Oskolkova O.V., Sarich N., Tian Y., Sitikov A., Tulapurkar M.E., Son S., Birukova A.A., Birukov K.G. (2017). Effects of prostaglandin lipid mediators on agonist-induced lung endothelial permeability and inflammation. Am. J. Physiol. Lung Cell. Mol. Physiol..

[168] Mitchell J.A., Shala F., Elghazouli Y., Warner T.D., Gaston-Massuet C., Crescente M., Armstrong P.C., Herschman H.R., Kirkby N.S. (2019). Cell-Specific Gene Deletion Reveals the Antithrombotic Function of COX1 and Explains the Vascular COX1/Prostacyclin Paradox. Circ. Res..

[169] Kirkby N.S., Lundberg M.H., Harrington L.S., Leadbeater P.D., Milne G.L., Potter C.M., Al-Yamani M., Adeyemi O., Warner T.D., Mitchell J.A. (2012). Cyclooxygenase-1, not cyclooxygenase-2, is responsible for physiological production of prostacyclin in the cardiovascular system. Proc. Natl. Acad. Sci. USA.

[170] Boegehold M.A. (2010). Endothelium-dependent control of vascular tone during early postnatal and juvenile growth. Microcirculation.

[171] Fukunaga K., Kohli P., Bonnans C., Fredenburgh L.E., Levy B.D. (2005). Cyclooxygenase 2 plays a pivotal role in the resolution of acute lung injury. J. Immunol..

[172] Huang T.-H., Fang P.-H., Li J.-M., Ling H.-Y., Lin C.-M., Shi C.-S. (2019). Cyclooxygenase-2 Activity Regulates Recruitment of VEGF-Secreting Ly6Chigh Monocytes in Ventilator-Induced Lung Injury. Int. J. Mol. Sci..

[173] Lee I.T., Yang C.M. (2013). Inflammatory signalings involved in airway and pulmonary diseases. Mediat. Inflamm..

[174] Peters T., Henry P.J. (2009). Protease-activated receptors and prostaglandins in inflammatory lung disease. Br. J. Pharmacol..

[175] Park G.Y., Christman J.W. (2006). Involvement of cyclooxygenase-2 and prostaglandins in the molecular pathogenesis of inflammatory lung diseases. Am. J. Physiol. Lung Cell. Mol. Physiol..

[176] Carey M.A., Germolec D.R., Langenbach R., Zeldin D.C. (2003). Cyclooxygenase enzymes in allergic inflammation and asthma. Prostaglandins Leukot. Essent. Fat. Acids.

[177] Kytölä J., Kääpä P., Uotila P. (2003). Meconium Aspiration Stimulates Cyclooxygenase-2 and Nitric Oxide Synthase-2 Expression in Rat Lungs. Pediatr. Res..

[178] Chen J.X., Berry L.C., Christman B.W., Meyrick B. (2003). Glutathione mediates LPS-stimulated COX-2 expression via early transient p42/44 MAPK activation. J. Cell. Physiol..

[179] Lee W., Kim J., Park E.K., Bae J.S. (2020). Maslinic Acid Ameliorates Inflammation via the Downregulation of NF-κB and STAT-1. Antioxidants.

[180] Szymanski K.V., Toennies M., Becher A., Fatykhova D., N’Guessan P.D., Gutbier B., Klauschen F., Neuschaefer-Rube F., Schneider P., Rueckert J. (2012). Streptococcus pneumoniae-induced regulation of cyclooxygenase-2 in human lung tissue. Eur. Respir. J..

[181] Bormann T., Maus R., Stolper J., Jonigk D., Welte T., Gauldie J., Kolb M., Maus U.A. (2021). Role of the COX2-PGE(2) axis in S. pneumoniae-induced exacerbation of experimental fibrosis. Am. J. Physiol. Lung Cell. Mol. Physiol..

[182] Chen J.S., Alfajaro M.M., Wei J., Chow R.D., Filler R.B., Eisenbarth S.C., Wilen C.B. (2020). Cyclooxgenase-2 is induced by SARS-CoV-2 infection but does not affect viral entry or replication. bioRxiv.

[183] Loo J., Spittle D.A., Newnham M. (2021). COVID-19, immunothrombosis and venous thromboembolism: Biological mechanisms. Thorax.

[184] Radi Z.A., Meyerholz D.K., Ackermann M.R. (2010). Pulmonary cyclooxygenase-1 (COX-1) and COX-2 cellular expression and distribution after respiratory syncytial virus and parainfluenza virus infection. Viral Immunol..

[185] Lee M.Y., Cheung C.Y., Peiris J.S. (2013). Role of cyclooxygenase-2 in H5N1 viral pathogenesis and the potential use of its inhibitors. Hong Kong Med. J..

[186] Shiraishi Y., Asano K., Niimi K., Fukunaga K., Wakaki M., Kagyo J., Takihara T., Ueda S., Nakajima T., Oguma T. (2008). Cyclooxygenase-2/Prostaglandin D2/CRTH2 Pathway Mediates Double-Stranded RNA-Induced Enhancement of Allergic Airway Inflammation. J. Immunol..

[187] Shirey K.A., Pletneva L.M., Puche A.C., Keegan A.D., Prince G.A., Blanco J.C., Vogel S.N. (2010). Control of RSV-induced lung injury by alternatively activated macrophages is IL-4R alpha-, TLR4-, and IFN-beta-dependent. Mucosal Immunol..

[188] Gopalakrishnan A., Joseph J., Shirey K.A., Keegan A.D., Boukhvalova M.S., Vogel S.N., Blanco J.C.G. (2022). Protection against influenza-induced Acute Lung Injury (ALI) by enhanced induction of M2a macrophages: Possible role of PPARγ/RXR ligands in IL-4-induced M2a macrophage differentiation. Front. Immunol..

[189] Kirkby N.S., Zaiss A.K., Wright W.R., Jiao J., Chan M.V., Warner T.D., Herschman H.R., Mitchell J.A. (2013). Differential COX-2 induction by viral and bacterial PAMPs: Consequences for cytokine and interferon responses and implications for anti-viral COX-2 directed therapies. Biochem. Biophys. Res. Commun..

[190] Sanches J.M., Rossato L., Lice I., Alves de Piloto Fernandes A.M., Bueno Duarte G.H., Rosini Silva A.A., de Melo Porcari A., de Oliveira Carvalho P., Gil C.D. (2021). The role of annexin A1 in Candida albicans and Candida auris infections in murine neutrophils. Microb. Pathog..

[191] Garth J.M., Reeder K.M., Godwin M.S., Mackel J.J., Dunaway C.W., Blackburn J.P., Steele C. (2017). IL-33 Signaling Regulates Innate IL-17A and IL-22 Production via Suppression of Prostaglandin E(2) during Lung Fungal Infection. J. Immunol..

[192] Zhou W., Zhang J., Toki S., Goleniewska K., Norlander A.E., Newcomb D.C., Wu P., Boyd K.L., Kita H., Peebles R.S. (2020). COX Inhibition Increases Alternaria-Induced Pulmonary Group 2 Innate Lymphoid Cell Responses and IL-33 Release in Mice. J. Immunol..

[193] Pereira P.A., Trindade B.C., Secatto A., Nicolete R., Peres-Buzalaf C., Ramos S.G., Sadikot R., Bitencourt Cda S., Faccioli L.H. (2013). Celecoxib improves host defense through prostaglandin inhibition during Histoplasma capsulatum infection. Mediat. Inflamm..

[194] Luh S.-P., Chiang C.-H. (2007). Acute lung injury/acute respiratory distress syndrome (ALI/ARDS): The mechanism, present strategies and future perspectives of therapies. J. Zhejiang Univ. Sci. B.

[195] Millar F.R., Summers C., Griffiths M.J., Toshner M.R., Proudfoot A.G. (2016). The pulmonary endothelium in acute respiratory distress syndrome: Insights and therapeutic opportunities. Thorax.

[196] Vassiliou A.G., Kotanidou A., Dimopoulou I., Orfanos S.E. (2020). Endothelial Damage in Acute Respiratory Distress Syndrome. Int. J. Mol. Sci..

[197] Sio S.W., Ang S.F., Lu J., Moochhala S., Bhatia M. (2010). Substance P upregulates cyclooxygenase-2 and prostaglandin E metabolite by activating ERK1/2 and NF-kappaB in a mouse model of burn-induced remote acute lung injury. J. Immunol..

[198] Ohmura T., Tian Y., Sarich N., Ke Y., Meliton A., Shah A.S., Andreasson K., Birukov K.G., Birukova A.A. (2017). Regulation of lung endothelial permeability and inflammatory responses by prostaglandin A2: Role of EP4 receptor. Mol. Biol. Cell.

[199] Perrot C.Y., Herrera J.L., Fournier-Goss A.E., Komatsu M. (2020). Prostaglandin E2 breaks down pericyte–endothelial cell interaction via EP1 and EP4-dependent downregulation of pericyte N-cadherin, connexin-43, and R-Ras. Sci. Rep..

[200] Kobayashi K., Horikami D., Omori K., Nakamura T., Yamazaki A., Maeda S., Murata T. (2016). Thromboxane A2 exacerbates acute lung injury via promoting edema formation. Sci. Rep..

[201] Mochizuki M., Ishii Y., Itoh K., Iizuka T., Morishima Y., Kimura T., Kiwamoto T., Matsuno Y., Hegab A.E., Nomura A. (2005). Role of 15-DeoxyΔ^12,14^ Prostaglandin J_2_ and Nrf2 Pathways in Protection against Acute Lung Injury. Am. J. Respir. Crit. Care Med..

[202] Kylhammar D., Rådegran G. (2017). The principal pathways involved in the in vivo modulation of hypoxic pulmonary vasoconstriction, pulmonary arterial remodelling and pulmonary hypertension. Acta Physiol..

[203] Liu C., Gao J., Chen B., Chen L., Belguise K., Yu W., Lu K., Wang X., Yi B. (2017). Cyclooxygenase-2 promotes pulmonary intravascular macrophage accumulation by exacerbating BMP signaling in rat experimental hepatopulmonary syndrome. Biochem. Pharmacol..

[204] Tang X., Liu C., Chen L., Yang Z., Belguise K., Wang X., Lu K., Yan H., Yi B. (2019). Cyclooxygenase-2 regulates HPS patient serum induced-directional collective HPMVEC migration via PKC/Rac signaling pathway. Gene.

[205] Smith W.L., DeWitt D.L., Garavito R.M. (2000). Cyclooxygenases: Structural, cellular, and molecular biology. Annu. Rev. Biochem..

[206] Marnett L.J. (2009). The COXIB experience: A look in the rearview mirror. Annu. Rev. Pharmacol. Toxicol..

[207] Marnett L.J. (2009). Mechanisms of cyclooxygenase-2 inhibition and cardiovascular side effects—The plot thickens. Cancer Prev. Res..

[208] Atukorala I., Hunter D.J. (2013). Valdecoxib: The rise and fall of a COX-2 inhibitor. Expert Opin. Pharmacother..

[209] Lebedeva E.S., Kuzubova N.N., Titova O.N., Surkova E.A. (2017). Effect of cyclooxygenase-2 inhibition on lung inflammation and hypoxia-inducible factor-1 signalling in COPD model. Eur. Respir. J..

[210] Chong S.J., Wong Y.C., Wu J., Tan M.H., Lu J., Moochhala S.M. (2014). Parecoxib reduces systemic inflammation and acute lung injury in burned animals with delayed fluid resuscitation. Int. J. Inflamm..

[211] Li A.M., Zhang L.N., Li W.Z. (2015). Amelioration of meconium-induced acute lung injury by parecoxib in a rabbit model. Int. J. Clin. Exp. Med..

[212] Robertson J.A., Sauer D., Gold J.A., Nonas S.A. (2012). The Role of Cyclooxygenase-2 in Mechanical Ventilation–Induced Lung Injury. Am. J. Respir. Cell. Mol. Biol..

[213] Meng F.Y., Gao W., Ju Y.N. (2017). Parecoxib reduced ventilation induced lung injury in acute respiratory distress syndrome. BMC Pharmacol. Toxicol..

[214] Zhang C., Hu S., Zosky G.R., Wei X., Shu S., Wang D., Chai X. (2021). Paracoxib Alleviates Ventilator-Induced Lung Injury Through Functional Modulation of Lung-Recruited CD11bloLy6Chi Monocytes. SHOCK.

[215] Qin J., Su X., Jin X., Zhao J. (2021). Parecoxib mitigates lung ischemia-reperfusion injury in rats by reducing oxidative stress and inflammation and up-regulating HO-1 expression. Acta Cir. Bras..

[216] Robb C.T., Goepp M., Rossi A.G., Yao C. (2020). Non-steroidal anti-inflammatory drugs, prostaglandins, and COVID-19. Br. J. Pharmacol..

[217] Verrall G.M. (2020). Scientific Rationale for a Bottom-Up Approach to Target the Host Response in Order to Try and Reduce the Numbers Presenting With Adult Respiratory Distress Syndrome Associated With COVID-19. Is There a Role for Statins and COX-2 Inhibitors in the Prevention and Early Treatment of the Disease?. Front. Immunol..

[218] Hoxha M. (2020). What about COVID-19 and arachidonic acid pathway?. Eur. J. Clin. Pharmacol..

[219] Baghaki S., Yalcin C.E., Baghaki H.S., Aydin S.Y., Daghan B., Yavuz E. (2020). COX2 inhibition in the treatment of COVID-19: Review of literature to propose repositioning of celecoxib for randomized controlled studies. Int. J. Infect. Dis..

[220] Hong W., Chen Y., You K., Tan S., Wu F., Tao J., Chen X., Zhang J., Xiong Y., Yuan F. (2020). Celebrex Adjuvant Therapy on Coronavirus Disease 2019: An Experimental Study. Front. Pharmacol..

[221] Shaban N.Z., Sleem A.A., Abu-Serie M.M., Maher A.M., Habashy N.H. (2022). Regulation of the NF-κB signaling pathway and IL-13 in asthmatic rats by aerosol inhalation of the combined active constituents of Punica granatum juice and peel. Biomed. Pharmacother..

